# Comparison of different ROI analysis methods for liver lesion characterization with simplified intravoxel incoherent motion (IVIM)

**DOI:** 10.1038/s41598-021-01108-6

**Published:** 2021-11-23

**Authors:** Narine Mesropyan, Petra Mürtz, Alois M. Sprinkart, Wolfgang Block, Julian A. Luetkens, Ulrike Attenberger, Claus C. Pieper

**Affiliations:** 1 Department of Diagnostic and Interventional Radiology, University Hospital Bonn, Venusberg-Campus 1, 53127 Bonn, Germany; 2 Department of Radiotherapy and Radiation Oncology, University Hospital Bonn, Venusberg-Campus 1, 53127 Bonn, Germany; 3 Department of Neuroradiology, University Hospital Bonn, Venusberg-Campus 1, 53127 Bonn, Germany

**Keywords:** Cancer, Cancer imaging

## Abstract

This study investigated the impact of different ROI placement and analysis methods on the diagnostic performance of simplified IVIM-DWI for differentiating liver lesions. 1.5/3.0-T DWI data from a respiratory-gated MRI sequence (b = 0, 50, 250, 800 s/mm^2^) were analyzed in patients with malignant (n = 74/54) and benign (n = 35/19) lesions. Apparent diffusion coefficient ADC = ADC(0,800) and IVIM parameters D_1_′ = ADC(50,800), D_2_′ = ADC(250,800), f_1_′ = f(0,50,800), f_2_′ = f(0,250,800), and D*' = D*(0,50,250,800) were calculated voxel-wise. For each lesion, a representative 2D-ROI, a 3D-ROI whole lesion, and a 3D-ROI from “good” slices were placed, including and excluding centrally deviating areas (CDA) if present, and analyzed with various histogram metrics. The diagnostic performance of 2D- and 3D-ROIs was not significantly different; e.g. AUC (ADC/D_1_′/f_1_′) were 0.958/0.902/0.622 for 2D- and 0.942/0.892/0.712 for whole lesion 3D-ROIs excluding CDA at 1.5 T (*p* > 0.05). For 2D- and 3D-ROIs, AUC (ADC/D_1_′/D_2_′) were significantly higher, when CDA were excluded. With CDA included, AUC (ADC/D_1_′/D_2_′/f_1_′/D*') improved when low percentiles were used instead of averages, and was then comparable to the results of average ROI analysis excluding CDA. For lesion differentiation the use of a representative 2D-ROI is sufficient. CDA should be excluded from ROIs by hand or automatically using low percentiles of diffusion coefficients.

## Introduction

Diffusion-weighted imaging (DWI) is one of the most promising non-contrast techniques that can be readily implemented in standard liver magnetic resonance imaging (MRI) examinations allowing for lesion detection and differentiation^1^. In routine clinical practice the apparent diffusion coefficient (ADC) is usually calculated with b-values between 0 and 500–1000 s/mm^2^ assuming a mono-exponential relationship between signal intensity and the b-value^2^. However the ADC is not only influenced by molecular diffusion, but also by other (pseudo) random motion such as blood flow in small vessels within the tissue (perfusion). According to the intravoxel incoherent motion (IVIM) theory, diffusion and perfusion effects can be separated assuming a bi-exponential behavior of signal intensity, ultimately yielding the diffusion coefficient D, the pseudo-diffusion coefficient D* and the perfusion fraction f^3–7^. f is associated with microvessel density^8,9^. D* was negatively correlated with the interstitial fluid pressure (IFP), which influences blood flow^10^. The problems with IVIM in clinical liver MRI are long acquisition times and limited data quality caused by respiratory and cardiac motion and by low signal-to-noise ratio, which may lead to unstable fitting results, measurement errors and poor reproducibility^11–14^. Improved stability can be achieved by segmented fitting approaches, which decrease the degree of freedom by determining the parameters step by step^15–19^ or by simplified IVIM, which uses numerically stable computation of IVIM parameter estimations from 4 b-values^20–27^.

For quantitative analysis of ADC and IVIM parameter maps in lesions a region of interest (ROI) based approach is the most commonly used^28–30^. However, there are different ROI-placement and analysis strategies, mostly only investigated for ADC: to place the ROIs into areas with most restricted diffusion (“hot spots”, focused ROIs), to average over multiple small ROIs placed into different regions, to place a large ROI on a central slice of a lesion, or to cover the whole lesion^7,21,23^. Usually ROI-analysis is done by averaging the voxel values within the ROI (mean). However, in order to address tumor heterogeneity, also histogram-based approaches are employed to subclassify different tumor diffusion and perfusion environments^7,31^.

The purpose of this study was to investigate whether there are differences in the diagnostic accuracy of ADC and IVIM parameters in the discrimination of liver lesions using different ROI placement and analysis strategies. We compared 2D- and 3D-volume ROIs, inclusion and exclusion of central necrosis, cystic components and scars, and ROI analysis by averaging and histogram metrics.

## Materials and methods

### Study cohort

**Figure 1:**
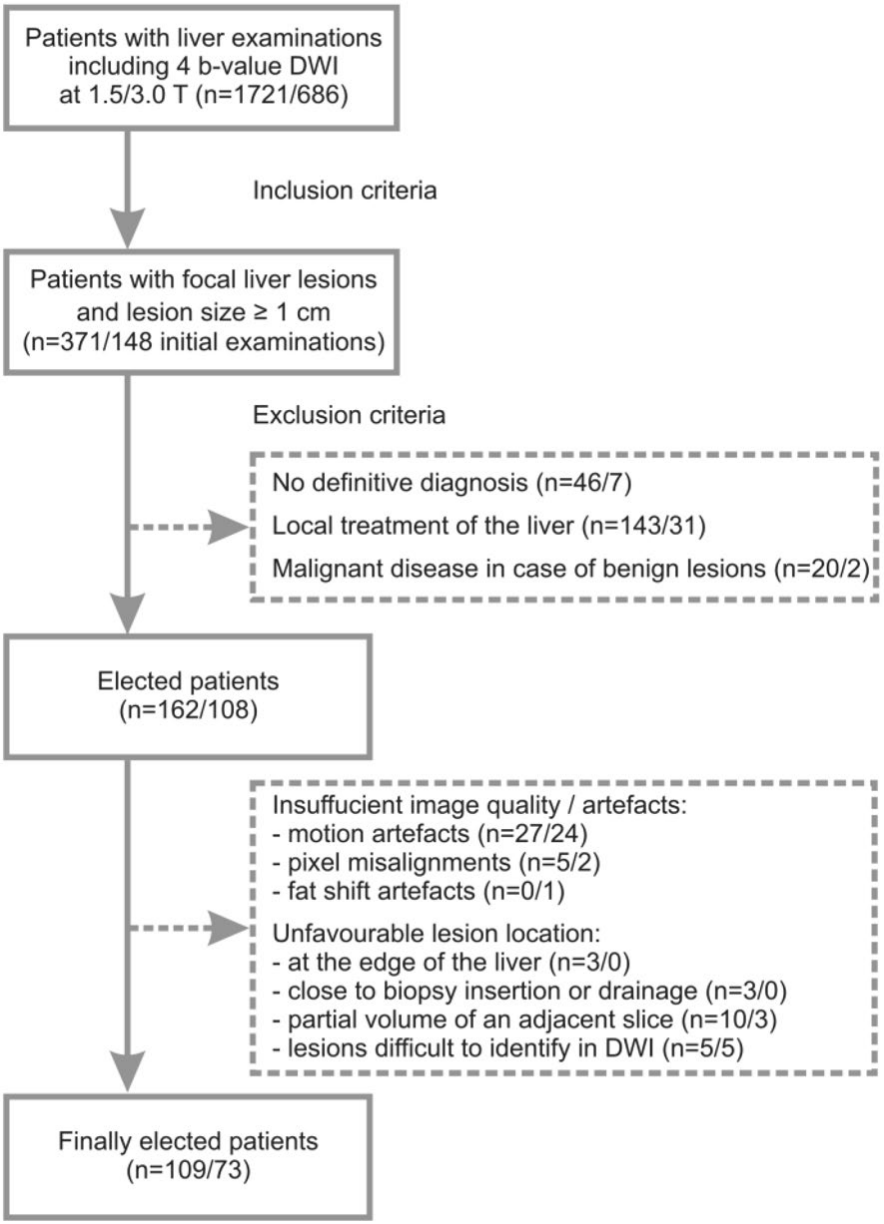
Flow chart of inclusion and exclusion criteria of the study sample.

This single-center retrospective study was approved by the ethics committee of the University Hospital of the Rheinische Friedrich-Wilhelms University Bonn, Germany, with a waiver for written informed consent. Data of consecutive patients with focal hepatic lesions ≥ 1 cm undergoing clinical MRI examination of the liver including 4 b-value DWI from 2013 to 2016 were used. A flowchart of patient inclusion and exclusion is given in Fig. 1. Finally, data of 109/73 patients at 1.5/3.0 T were analyzed (Table 1). These two patient groups had already been examined in previous studies^21,23^. In those studies basic investigations concerning simplified IVIM for liver lesion characterization had been performed. In the present study, the data were used to investigate the influence of different ROI placement and analysis methods concerning diagnostic accuracy.

**Table 1:** Group composition and demographic data of included subjects at 3.0 and 1.5 T.

Patients	3.0 T				1.5 T			
	Total number	Number of males	Age (MV ± SD) [years]	Age range [years]	Total number	Number of males	Age (MV ± SD) [years]	Age range [years]
HCC	26	23	69 ± 10	50–87	32	20	71 ± 9	55–87
CCC	5	3	72 ± 3	68–76	8	4	69 ± 10	57–85
CRC	13	8	63 ± 8	52–81	22	17	60 ± 10	47–87
BC	10	0	57 ± 9	45–72	12	0	60 ± 6	48–70
Hemangioma	11	5	46 ± 13	32–72	23	12	51 ± 14	34–84
FNH	8	0	37 ± 11	22–49	12	1	37 ± 13	14–54

MV—mean value, SD—standard deviation, HCC—hepatocellular carcinoma, CCC—cholangiocellular carcinoma, CRC—metastases of colorectal carcinoma, BC—metastases of breast cancer, FNH—focal nodular hyperplasia.

Diagnosis of liver lesions was undertaken within clinical routine. Cholangiocellular carcinomas (CCCs) were histologically proven. Hepatocellular carcinomas (HCCs) were either histologically proven or diagnosed according to the American Association for the Study for Liver Disease MRI criteria^32^. Diagnosis of metastasis was based on typical imaging features in combination with histologically proven primary cancer. Diagnosis of focal nodular hyperplasia (FNH) or haemangioma was established on the basis of typical radiological findings on contrast-enhanced MRI and was confirmed by at least one follow-up examination.

### Magnetic resonance imaging

Imaging was performed on clinical whole-body 1.5/3.0-T MRI systems (Ingenia, Philips Healthcare; 1.5/3.0-T gradient system: 45/45 mT/m maximum amplitude, 200/200 T/m/s maximum slew rate; 3.0-T system with dual source RF transmission) using 32-channel abdominal coils with a digital interface for signal reception. The standardized imaging protocol included a DWI sequence with a respiratory-triggered single-shot spin-echo echo-planar imaging variant with four b-values (0, 50, 250, 800 s/mm^2^) before contrast agent administration (Table 2). For each slice, an isotropic diffusion-weighted image was reconstructed from the three images obtained for the different diffusion directions.

**Table 2:** Parameters of the diffusion-weighted imaging (DWI) sequence.

Name	Value at 3.0 T	Value at 1.5 T
FOV (RLxAP)/orientation	400 × 352 mm/transversal	380 × 326 mm/transversal
Slice number/thickness/gap	26/7.0 mm/0.7 mm	30/7.0 mm/0.7 mm
Matrix/resolution	132 × 113/3.0 × 3.1 mm	112 × 94/3.4 × 3.5 mm
Echo time (TE)	44 ms	63 ms
Repetition time (TR)	1 respiratory cycle	1 respiratory cycle
Imaging time per respiration	1894 ms	1600 ms
EPI-/half-Fourier-/SENSE-factor	41/0.6/3	51/0.6/2
Diffusion gradients	3 orthogonal directions	3 orthogonal directions
b-values (number of averages per direction)	0, 50, 250 s/mm^2^ (NSA = 2), 800 s/mm^2^ (NSA = 4)	0, 50, 250 s/mm^2^ (NSA = 2), 800 s/mm^2^ (NSA = 4)
Fat suppression methods	SPIR + SSGR	SPIR
Water-fat shift/BW	11.1 Pixel/39.0 Hz	9.2 Pixel/23.6 Hz
BW in EPI frequency direction	3346.0 Hz	1437.9 Hz
Acquisition time	Around 4 min (2:42 min without gating)	Around 4 min (2:42 min without gating)

SENSE—parallel imaging with sensitivity encoding, FOV—field of view, RL—right-left, AP—anterior–posterior, EPI—echo-planar imaging, SPIR—spectral presaturation by inversion recovery, SSGR—slice-selective gradient reversal (uses slice-selection gradients of opposite polarity for the 180° pulses taking advantage of the chemical shift of fat with respect to water), BW—bandwidth.

### Postprocessing

As described previously^21,23^, two different approximations of D and f were calculated from signal intensities S(b) and S(0) of the acquired b-values, one from b_0_ = 0, b_1_ = 50, b_3_ = 800 and one from b_0_ = 0, b_2_ = 250, b_3_ = 800 s/mm^2^:

1 $$D_{1} ^{\prime} = ADC(50,800) = \frac{{\ln (S(b_{1} )) - \ln (S(b_{3} ))}}{{b_{3} - b_{1} }}$$

2 $$D_{2} ^{\prime} = ADC(250,800) = \frac{{\ln (S(b_{2} )) - \ln (S(b_{3} ))}}{{b_{3} - b_{2} }}$$

3 $$f_{1} ^{\prime} = f(0,50,800) = 1 - \frac{{S(b_{1} )}}{S(0)} \cdot \exp^{{D_{1} ^{\prime} \cdot b_{1} }}$$

4 $$f_{2} ^{\prime} = f(0,250,800) = 1 - \frac{{S(b_{2} )}}{S(0)} \cdot \exp^{{D_{2} ^{\prime} \cdot b_{2} }}$$

From the four b-values, D* was approximated by using D_2_′ and f_2_′ and the reading for b_1_:

5 $$D^{*\prime} = D^{*}(0,50,250,800) = - \frac{1}{{b_{1} }} \cdot \ln \left[ {\frac{1}{{f_{2} ^{\prime}}} \cdot \left( {\frac{{S(b_{1} )}}{S(0)} - \left( {1 - f_{2} ^{\prime}} \right) \cdot \exp^{{ - D_{2} ^{\prime} \cdot b_{1} }} } \right)} \right]$$

D*′ cannot be determined for all voxels, because some voxels are not affected by perfusion. Voxels with not defined values were excluded from ROI analysis.

Moreover, the conventional ADC was calculated:

6 $$ADC = ADC(0,800) = \frac{{\ln (S(b_{0} )) - \ln (S(b_{3} ))}}{{b_{3} - b_{0} }}$$

Parameter maps and ROI analyses were calculated offline using custom written software in MATLAB (MathWorks, Natick, MA).

### Image analysis

Image analysis was performed by a radiologist (N.M.) with 3 years of experience and checked by a radiologist (C.C.P.) with 10 years of experience in abdominal imaging and a physicist (P.M.) with more than 20 years of experience in DWI. All were blinded to clinical information.

One reference lesion per lesion type was analyzed. For each included lesion, 2D- and 3D-volume ROI-based analyses were performed. ROIs were placed as large as possible using DWI with highest contrast between lesion and normal tissue and excluding areas close to the lesion rim to avoid partial-volume effects. After the anatomical position of each ROI had been visually cross-checked for pixel misalignments between images with different b-values, the ROI was analyzed in the related parameter maps.

**Figure 2:**
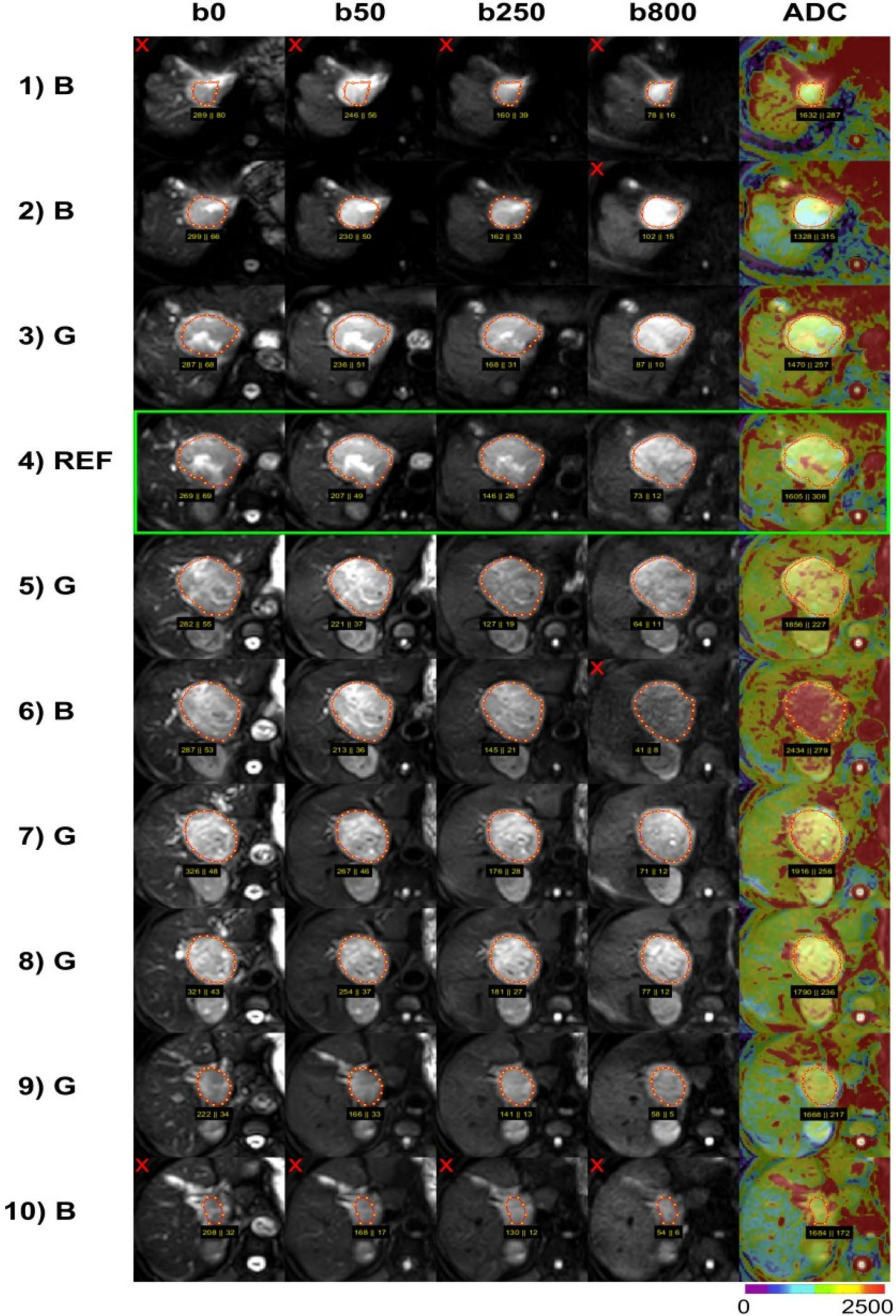
A typical example of 2D and 3D DWI IVIM analysis in a hepatocellular carcinoma at 1.5 T. Original diffusion-weighted images with b = 0, 50, 250, 800 s/mm^2^ are presented together with conventional ADC maps displayed as color-coded overlays over b800 images. For analysis, on each tumor-containing slice a region of interest (ROI) was selected, where ADC and IVIM parameters (not shown) were analyzed. ADC values are given in units of 10^−6^ mm^2^/s. Slices largely unaffected by artifacts were defined as good (“G”), slices close to the lesion’s rim (partial volume) or with images affected by artifacts (see red x) due to motion, susceptibility or pixel misalignments were defined as bad (“B”). One central “good” slice served as reference (“REF”) for the 2D analysis (see green frame), hereby slices in the lower part of the liver should be preferred due to lower motion influences from the heart. For 3D analysis, the voxels of the 2D ROI were combined with voxels of the ROIs on other “good” slices (3DG), voxels of all ROIs was used for whole lesion analysis (3DA).

For 2D-analysis, one hand-drawn ROI was placed centrally in each lesion on a single representative slice (reference slice), which was largely unaffected by motion and susceptibility artifacts and pixel misalignments. For the 3D-volume analysis, a hand-drawn ROI was placed on each slice of the lesion. Slices with artifacts and pixel misalignments as well as the first and the last slice (due to potential partial volume effect) were marked as “bad”. An example of ROI placement is given in Fig. 2. Data from all slices (“good” and “bad”) were combined into a whole-lesion 3D-volume ROI (3DA). Furthermore, a second 3D-volume ROI was calculated including only the “good” slices (3DG). Thus, in each lesion three different ROI-sizes were investigated (2D, 3DA, 3DG).

**Figure 3:**
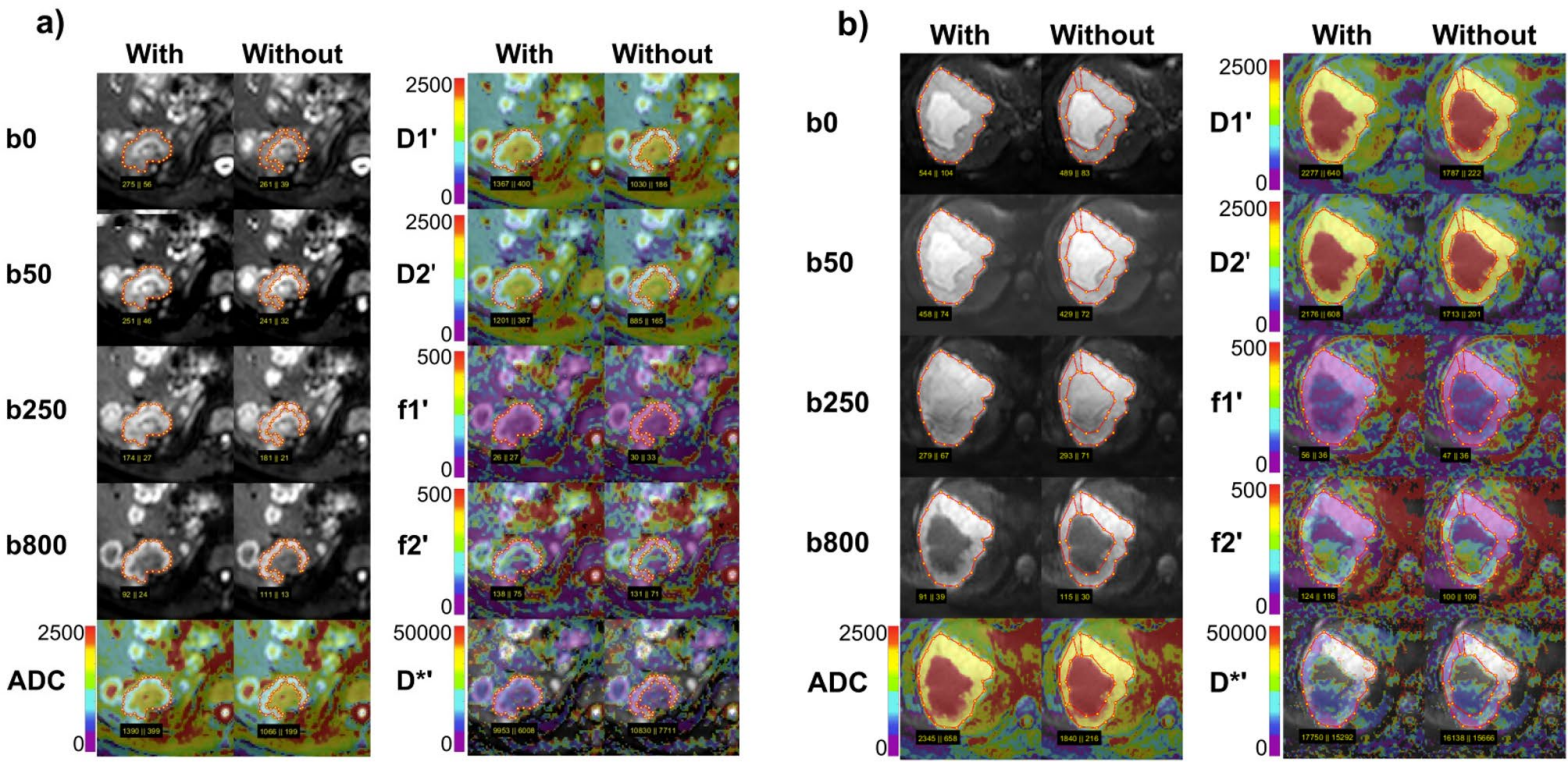
Typical examples of DWI IVIM analysis comparing in- and exclusion of necrosis in a metastasis of colorectal carcinoma (**a**) and of liquid in a hemangioma (**b**) at 1.5 T. For one central slice per lesion, original diffusion-weighted images with b = 0, 50, 250, 800 s/mm^2^ are presented together with conventional ADC, diffusion sensitive D_1_′ and D_2_′ parameter maps, and perfusion sensitive f_1_′, f_2_′, D*′ parameter maps. The parameter maps are displayed as color-coded overlays over b = 800. Values of ADC, D_1_′, D_2_′ and D*′ are given in units of 10^–6^ mm^2^/s, those of f_1_′ and f_2_′ in 10^−3^. If bad data quality led to negative parameter values or to not defined values, these voxels were not colorized. When necrosis/cystic components were excluded (“Without”) from regions of interests (ROIs), the diffusion sensitive parameters were significantly lower compared to inclusion (“With”). Perfusion sensitive parameters remained unchanged because there is only low perfusion in the metastasis and hemangioma anyway.

For lesions with central necrosis, cystic components or scars (centrally deviating areas in DWI), the 2D- and 3D-ROI placements were repeated with exclusion of such areas. Two example analyses are given in Fig. 3. These measurements allowed the evaluation of different ROI sizes as well as of different lesion tissues included to the ROIs.

Finally, a histogram analysis was performed for each 2D-ROI. The following histogram metrics were calculated: median, standard deviation, the 5th, 10th, 25th, 75th, 90th, 95th percentiles, skewness and kurtosis.

### Statistical analysis

Statistical analysis was performed using SPSS (Version 24.0, IBM) and pROC package (Version 1.16.2) in R (Version 3.6.1)^33^. Receiver operating characteristic (ROC) analysis was performed for liver lesions discrimination. Youden’s index was used to determine the optimal cut-off of the ROC curve providing the best trade-off between sensitivity and specificity. DeLong method was used to compare dependent ROC curves^34^. The area under the curve (AUC) based on mean ROI values was compared for the different ROI variants. Furthermore, it was investigated, whether AUC values can be improved by using one of the histogram metrics instead of the mean value. These investigations were carried out for both types of ROIs, including and excluding centrally deviating areas. In order to investigate whether histogram analyses may replace manual exclusion of such areas, additionally a comparison was performed using ROIs excluding such areas in case of mean values and including them in case of histogram metrics.

### Ethical approval and informed consent

The presented study was approved by the institutional review board of the University of Bonn and hence all methods were performed in compliance with the ethical standards set in the 1964 Declaration of Helsinki as well as its later amendments. Written informed consent was waived.

## Results

**Figure 4:**
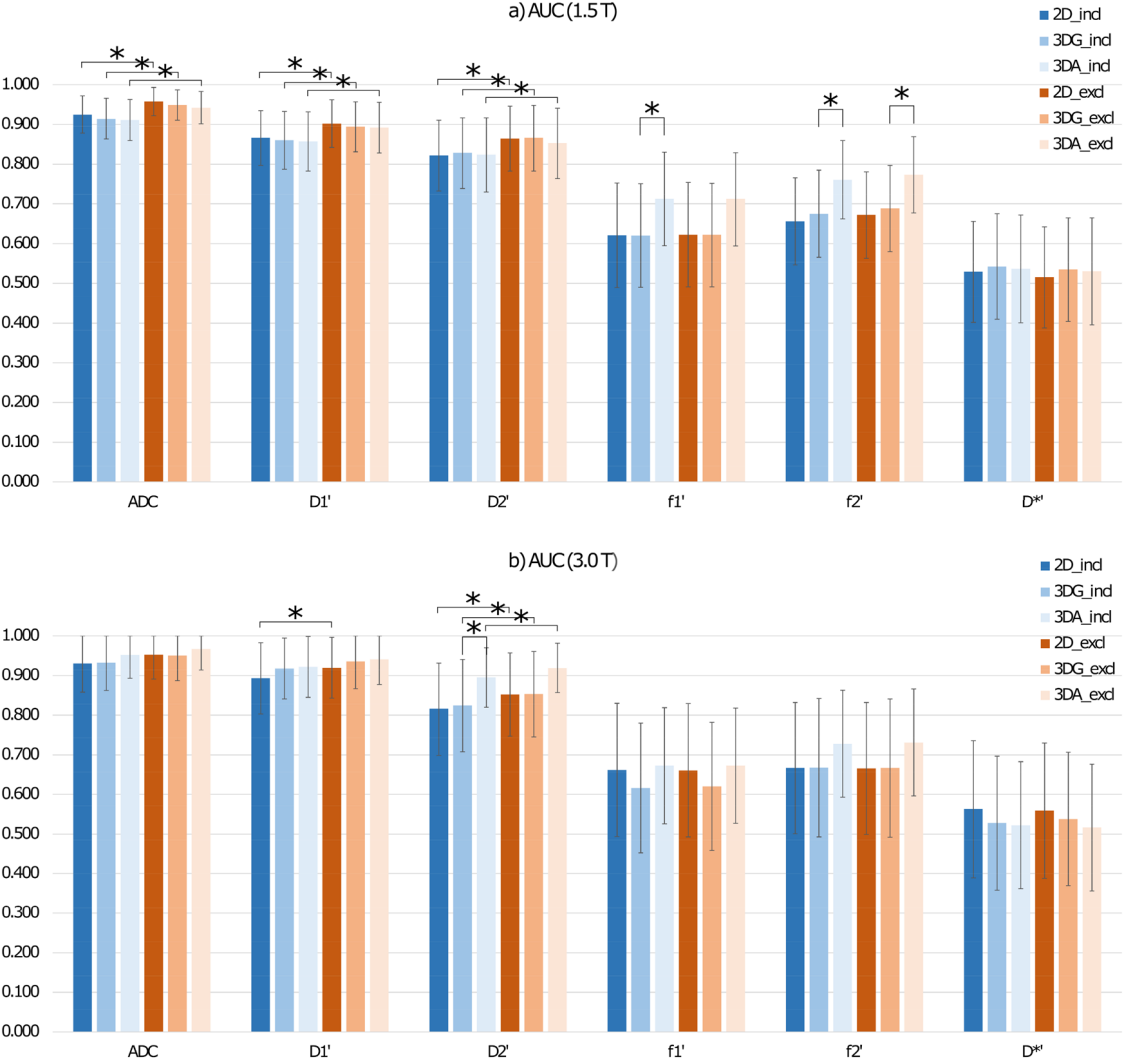
Overview to obtained AUC values (**a**) at 1.5 T and (**b**) at 3.0 T for the different ROIs (2D, 3DG, 3DA) and with included and excluded central necrosis, cystic components or scars. Significant differences are marked by “*”.

At 1.5/3.0 T, 74/54 malignant and 35/19 benign liver lesions were analyzed (Table 1). Mean volume of malignant lesions was 96.6/76.6 cm^3^ (range: 1.3–1715.7/1.2–521.2 cm^3^) and of benign lesions 72.1/20.4 cm^3^ (range: 0.9–856.3/1.1–118.3 cm^3^). Of these 109/73 lesions, 36/11 had centrally deviating areas. In total, 1333 ROIs were placed. The mean values of ADC and IVIM parameters for the benign and malignant lesion group together with the ROC analyses results for lesion differentiation are presented in Table 3. In Fig. 4 an overview to the obtained AUC values are given. In general, the values of diffusion and perfusion sensitive parameters were lower in malignant lesions than in benign lesions.

**Table 3:** Results of ADC and IVIM parameter value analysis within different regions of interest (ROIs) and receiver operating characteristic (ROC) analysis of benign and malignant liver lesions.

ROI	Par	Malignant			Benign			Dir	AUC	CI1	CI2	Cut-off	Sen	Spec	Acc
		MV	SD	N	MV	SD	N								
**(a) 1.5 T**															
**ROIs including centrally deviating areas**															
2D	ADC	1182	216	74	1712	329	35	>	0.925	0.878	0.972	1335.8	0.797	0.914	0.835
	D_1_′	1115	224	74	1600	401	35	>	0.866	0.796	0.935	1130.4	0.622	0.943	0.725
	D_2_′	990	280	74	1442	433	35	>	0.822	0.733	0.911	1105.0	0.689	0.857	0.743
	f_1_′	64	31	74	97	70	35	>	0.621	0.490	0.753	110.7	0.905	0.457	0.761
	f_2_′	145	96	74	191	104	35	>	0.656	0.546	0.766	198.9	0.838	0.429	0.706
	D*′	18,370	8332	74	21,200	13,245	35	>	0.529	0.401	0.656	25,008.0	0.811	0.371	0.670
3DG	ADC	1202	223	74	1731	356	35	>	0.914	0.863	0.966	1311.7	0.730	0.943	0.798
	D_1_′	1129	226	74	1616	401	35	>	0.860	0.787	0.933	1431.5	0.919	0.657	0.835
	D_2_′	1020	253	74	1467	436	35	>	0.828	0.739	0.917	1183.2	0.797	0.743	0.780
	f_1_′	66	29	74	99	63	35	>	0.620	0.490	0.751	106.5	0.932	0.457	0.780
	f_2_′	139	68	74	192	97	35	>	0.675	0.566	0.785	183.5	0.838	0.457	0.716
	D*′	17,436	5452	74	19,242	8748	35	>	0.542	0.410	0.675	24,886.1	0.932	0.371	0.752
3DA	ADC	1230	234	74	1748	329	35	>	0.911	0.859	0.963	1498.4	0.892	0.771	0.853
	D_1_′	1147	235	74	1607	360	35	>	0.857	0.783	0.932	1468.4	0.919	0.686	0.844
	D_2_′	1057	246	74	1466	375	35	>	0.824	0.730	0.917	1206.9	0.824	0.771	0.807
	f_1_′	73	29	74	115	56	35	>	0.712	0.595	0.830	117.3	0.932	0.514	0.798
	f_2_′	135	55	74	202	87	35	>	0.761	0.662	0.859	172.4	0.851	0.657	0.789
	D*′	18,120	4533	74	19,437	7967	35	>	0.536	0.401	0.672	24,541.3	0.946	0.343	0.752
**ROIs excluding centrally deviating areas**															
2D	ADC	1124	180	74	1692	313	35	>	0.958	0.922	0.993	1338.5	0.892	0.914	0.899
	D_1_′	1057	188	74	1580	387	35	>	0.902	0.842	0.962	1173.6	0.757	0.886	0.798
	D_2_′	939	250	74	1423	416	35	>	0.864	0.783	0.946	1142.5	0.838	0.829	0.835
	f_1_′	63	31	74	97	70	35	>	0.622	0.491	0.754	114.5	0.932	0.457	0.780
	f_2_′	141	96	74	191	104	35	>	0.672	0.563	0.781	140.0	0.622	0.657	0.633
	D*′	18,837	8603	74	21,189	13,251	35	>	0.515	0.388	0.642	24,996.2	0.784	0.371	0.651
3DG	ADC	1144	187	74	1717	357	35	>	0.949	0.911	0.987	1310.2	0.838	0.943	0.872
	D_1_′	1072	194	74	1602	399	35	>	0.894	0.831	0.957	1333.0	0.946	0.714	0.872
	D_2_′	966	215	74	1454	432	35	>	0.866	0.783	0.948	1179.3	0.892	0.743	0.844
	f_1_′	66	29	74	99	63	35	>	0.622	0.491	0.752	106.8	0.932	0.457	0.780
	f_2_′	137	66	74	192	97	35	>	0.688	0.580	0.797	149.9	0.703	0.629	0.679
	D*′	17,634	5757	74	19,225	8735	35	>	0.535	0.404	0.665	24,616.8	0.905	0.371	0.734
3DA	ADC	1176	201	74	1736	330	35	>	0.942	0.902	0.983	1447.8	0.932	0.800	0.890
	D_1_′	1094	203	74	1594	357	35	>	0.892	0.828	0.956	1314.9	0.905	0.743	0.853
	D_2_′	1006	211	74	1454	371	35	>	0.853	0.764	0.941	1314.9	0.946	0.714	0.872
	f_1_′	73	30	74	115	56	35	>	0.712	0.594	0.829	116.9	0.919	0.514	0.789
	f_2_′	134	55	74	202	87	35	>	0.773	0.677	0.869	172.2	0.865	0.657	0.798
	D*′	18,277	4901	74	19,381	7927	35	>	0.530	0.396	0.665	24,767.8	0.932	0.343	0.743
**(b) 3.0 T**															
**ROIs including centrally deviating areas**															
2D	ADC	1120	183	54	1566	251	19	>	0.931	0.858	1.000	1419.4	0.963	0.789	0.918
	D_1_′	1062	175	54	1463	278	19	>	0.893	0.803	0.983	1292.6	0.926	0.737	0.877
	D_2_′	976	189	54	1310	318	19	>	0.816	0.699	0.932	1183.8	0.870	0.632	0.808
	f_1_′	59	39	54	98	66	19	>	0.662	0.494	0.830	96.6	0.870	0.526	0.781
	f_2_′	118	76	54	188	118	19	>	0.667	0.501	0.832	172.1	0.833	0.579	0.767
	D*′	17,273	7256	53	19,740	10,820	17	>	0.563	0.389	0.736	21,309.9	0.774	0.412	0.686
3DG	ADC	1138	181	54	1549	224	19	>	0.933	0.862	1.000	1420.9	0.963	0.789	0.918
	D_1_′	1081	175	54	1477	229	19	>	0.918	0.841	0.995	1392.7	0.981	0.737	0.918
	D_2_′	1000	166	54	1328	307	19	>	0.825	0.708	0.941	1345.9	1.000	0.526	0.877
	f_1_′	63	39	54	92	64	19	>	0.616	0.452	0.780	125.4	0.944	0.368	0.795
	f_2_′	118	52	54	183	116	19	>	0.668	0.493	0.842	180.0	0.852	0.526	0.767
	D*′	17,477	6597	54	19,055	10,602	18	>	0.528	0.358	0.697	34,209.4	0.981	0.167	0.778
3DA	ADC	1148	173	54	1578	209	19	>	0.952	0.893	1.000	1391.4	0.944	0.895	0.932
	D_1_′	1088	168	54	1489	223	19	>	0.922	0.845	0.999	1383.9	0.981	0.789	0.932
	D_2_′	1016	159	54	1358	241	19	>	0.895	0.820	0.970	1067.5	0.630	1.000	0.726
	f_1_′	66	39	54	96	46	19	>	0.673	0.526	0.819	83.5	0.759	0.579	0.712
	f_2_′	119	55	54	179	81	19	>	0.728	0.593	0.863	125.8	0.648	0.789	0.685
	D*′	17,457	5301	54	17,501	8499	19	<	0.522	0.362	0.683	17,598.3	0.537	0.632	0.562
**ROIs excluding centrally deviating areas**															
2D	ADC	1090	167	54	1566	251	19	>	0.953	0.891	1.000	1276.1	0.870	0.947	0.890
	D_1_′	1032	156	54	1463	278	19	>	0.920	0.843	0.997	1214.9	0.944	0.789	0.904
	D_2_′	945	171	54	1310	318	19	>	0.852	0.747	0.957	1183.8	0.963	0.632	0.877
	f_1_′	59	38	54	98	66	19	>	0.661	0.493	0.829	96.6	0.870	0.526	0.781
	f_2_′	119	76	54	188	118	19	>	0.666	0.499	0.832	172.1	0.833	0.579	0.767
	D*′	17,895	8443	54	19,740	10,820	17	>	0.559	0.388	0.730	21,309.9	0.759	0.412	0.676
3DG	ADC	1110	163	54	1549	224	19	>	0.951	0.887	1.000	1283.1	0.852	0.947	0.877
	D_1_′	1053	156	54	1477	229	19	>	0.936	0.867	1.000	1334.1	1.000	0.789	0.945
	D_2_′	974	149	54	1328	307	19	>	0.853	0.745	0.961	1182.9	0.926	0.632	0.849
	f_1_′	63	39	54	92	64	19	>	0.620	0.458	0.782	125.4	0.944	0.368	0.795
	f_2_′	118	52	54	183	116	19	>	0.667	0.492	0.841	178.1	0.852	0.526	0.767
	D*′	17,301	6543	54	19,055	10,602	18	>	0.538	0.370	0.707	16,348.6	0.500	0.667	0.542
3DA	ADC	1125	160	54	1578	209	19	>	0.967	0.914	1.000	1386.6	0.981	0.895	0.959
	D_1_′	1065	154	54	1489	223	19	>	0.941	0.877	1.000	1367.4	1.000	0.789	0.945
	D_2_′	995	150	54	1358	241	19	>	0.919	0.857	0.982	1067.5	0.704	1.000	0.781
	f_1_′	67	39	54	96	46	19	>	0.673	0.527	0.818	116.1	0.907	0.421	0.781
	f_2_′	118	54	54	179	81	19	>	0.731	0.596	0.866	126.7	0.648	0.789	0.685
	D*′	17,394	5200	54	17,501	8499	19	<	0.517	0.356	0.677	17,598.3	0.519	0.632	0.548

ADC, D_1_', D_2_', D*' values are given in units of 10^−6^ mm^2^/s, f_1_′ and f_2_' values are given in units of 10^−3^.

The highest AUC values for lesion differentiation were found for ADC (0.967–0.911) and D_1_′ (0.941–0.857) followed by D_2_′ (0.919–0.816), f_2_′ (0.731–0.656), f_1_′ (0.673–0.616), and D*′ (0.563–0.515). For all parameters, diagnostic performance was compared for the different 2D- and 3D-ROI variants, for ROIs in- and excluding centrally deviating areas, and for mean values and histogram metrics.

### Comparison of 2D- and 3D-ROIs

In Table 4 the results of the AUC value comparisons with respect to the different ROI types (2D, 3DG, 3DA) are presented. No significant differences were found in any of the comparisons, neither for ROIs that include centrally deviating areas, nor for those excluding such areas. The only exceptions were that AUC values for 3DA ROIs compared to those for 3DG ROIs were slightly larger in case of f_1_′ and f_2_′ at 1.5 T (for ROIs including centrally deviating areas: 0.712 vs 0.620 with *p* = 0.049 and 0.761 vs 0.675 with *p* = 0.031, respectively; for ROIs excluding those areas: 0.712 vs 0.622 with *p* = 0.055 and 0.773 vs 0.688 with *p* = 0.029, respectively), and in case of D_2_′ at 3.0 T, but only for ROIs including centrally deviating areas (0.895 vs 0.825 with *p* = 0.029).

**Table 4:** Comparison of AUC values of the ROC curves obtained from 2 and 3D ROIs (see Table 2) at 1.5 T (a) and 3.0 T (b).

Par	AUC 2D	AUC 3DG	*P*	AUC 2D	AUC 3DA	*P*	AUC 3DG	AUC 3DA	*P*
**(a) 1.5 T**									
**ROIs including centrally deviating areas**									
ADC	0.925	0.914	0.358	0.925	0.911	0.372	0.914	0.911	0.751
D_1_′	0.866	0.860	0.696	0.866	0.857	0.631	0.860	0.857	0.783
D_2_′	0.822	0.828	0.817	0.822	0.824	0.959	0.828	0.824	0.756
f_1_′	0.621	0.620	0.986	0.621	0.712	0.137	0.620	0.712	0.049*
f_2_′	0.656	0.675	0.689	0.656	0.761	0.071	0.675	0.761	0.031*
D*′	0.529	0.542	0.724	0.529	0.536	0.877	0.542	0.536	0.861
**ROIs excluding centrally deviating areas**									
ADC	0.958	0.949	0.291	0.958	0.942	0.224	0.949	0.942	0.394
D_1_′	0.902	0.894	0.594	0.902	0.892	0.565	0.894	0.892	0.815
D_2_′	0.864	0.866	0.961	0.864	0.853	0.663	0.866	0.853	0.379
f_1_′	0.622	0.622	0.986	0.622	0.712	0.143	0.622	0.712	0.055
f_2_′	0.672	0.688	0.729	0.672	0.773	0.075	0.688	0.773	0.029*
D*′	0.515	0.535	0.608	0.515	0.530	0.755	0.535	0.530	0.896
**(b) 3.0 T**									
**ROIs including centrally deviating areas**									
ADC	0.931	0.933	0.904	0.931	0.952	0.106	0.933	0.952	0.167
D_1_′	0.893	0.918	0.267	0.893	0.922	0.223	0.918	0.922	0.715
D_2_′	0.816	0.825	0.803	0.816	0.895	0.056	0.825	0.895	0.029*
f_1_′	0.662	0.616	0.299	0.662	0.673	0.851	0.616	0.673	0.254
f_2_′	0.667	0.668	0.988	0.667	0.728	0.444	0.668	0.728	0.280
D*′	0.563	0.513	0.374	0.563	0.518	0.780	0.528	0.498	0.848
**ROIs excluding centrally deviating areas**									
ADC	0.953	0.951	0.867	0.953	0.967	0.174	0.951	0.967	0.186
D_1_′	0.920	0.936	0.461	0.920	0.941	0.349	0.936	0.941	0.669
D_2_′	0.852	0.853	0.975	0.852	0.919	0.100	0.853	0.919	0.059
f_1_′	0.661	0.620	0.338	0.661	0.673	0.837	0.620	0.673	0.282
f_2_′	0.666	0.667	0.988	0.666	0.731	0.416	0.667	0.731	0.243
D*′	0.559	0.528	0.591	0.559	0.504	0.720	0.538	0.492	0.766

AUC—area under the curve,

*marks significant results, *P*—*p*-value.

### Comparison of ROIs with included and excluded central necrosis, cystic components or scars

Table 5 summarizes the results of AUC value comparison with respect to included tissue. Exclusion of centrally deviating areas from ROIs yields larger AUC values of ADC, D_1_′, and D_2_′, for all 2D- and 3D-ROI variants. Improvements were significant at 1.5 T, at 3 T, however, sometimes only by tendency, potentially due to fewer cases with centrally deviating areas. For 2D-ROIs at 1.5 T for example, AUC values of ADC improved from 0.925 to 0.958 (*p* = 0.01), of D_1_′ from 0.866 to 0.902 (*p* = 0.0081), and of D_2_′ from 0.822 to 0.864 (0.00089). Perfusion parameters did not show any differences. Typical examples of DWI IVIM analysis comparing in- and exclusion of centrally deviating areas are presented in Fig. 3.

**Table 5:** Comparison of AUC values of the ROC curves obtained from ROIs including (incl) and excluding (excl) centrally deviating areas like necrosis, cystic components or scars (see Table 1) at 1.5 T (a) and 3.0 T (b).

Parameter	ROI	AUC(incl)	AUC(excl)	*P*
**(a) 1.5 T**				
ADC	2D	0.925	0.958	1.0E−02*
	3DG	0.914	0.949	4.8E−03*
	3DA	0.911	0.942	4.8E−03*
D_1_′	2D	0.866	0.902	8.1E−03*
	3DG	0.860	0.894	1.1E−03*
	3DA	0.857	0.892	6.8E−04*
D_2_′	2D	0.822	0.864	8.9E−04*
	3DG	0.828	0.866	5.6E−04*
	3DA	0.824	0.853	7.4E−04*
f_1_′	2D	0.621	0.622	8.6E−01
	3DG	0.620	0.622	7.6E−01
	3DA	0.712	0.712	8.6E−01
f_2_′	2D	0.656	0.672	2.2E−01
	3DG	0.675	0.688	9.0E−02
	3DA	0.761	0.773	5.8E−02
D*′	2D	0.529	0.515	1.8E−01
	3DG	0.542	0.535	2.3E−01
	3DA	0.536	0.530	3.6E−01
**(b) 3.0 T**				
ADC	2D	0.931	0.953	0.068
	3DG	0.933	0.951	0.069
	3DA	0.952	0.967	0.102
D_1_′	2D	0.893	0.920	0.026*
	3DG	0.918	0.936	0.069
	3DA	0.922	0.941	0.052
D_2_′	2D	0.816	0.852	0.012*
	3DG	0.825	0.853	0.021*
	3DA	0.895	0.919	0.033*
f_1_′	2D	0.662	0.661	0.727
	3DG	0.616	0.620	0.505
	3DA	0.673	0.673	1.000
f_2_′	2D	0.667	0.666	0.805
	3DG	0.668	0.667	0.816
	3DA	0.728	0.731	0.420
D*′	2D	0.563	0.569	0.199
	3DG	0.528	0.538	0.174
	3DA	0.522	0.517	0.465

AUC—area under the curve.

*marks significant results, *P*—*p* value.

### Comparison of mean values versus histogram analysis

Table S1 gives the mean values and values of histogram metrics for the benign and malignant lesion group together with the ROC analyses results for lesion differentiation using 2D-ROIs. In Table S2 the results of the different AUC value comparisons are given.

At 1.5 T, the 5th and 10th percentiles of ADC and D_1_′ and the 25th percentiles of ADC, D_1_′ and D_2_′ lead to significantly higher AUC values than the mean values for ROIs including centrally deviating areas. For example, by using the 10th percentile instead of mean value, AUC values could be improved for ADC from 0.925 to 0.969 (*p* = 0.018), for D_1_′ from 0.866 to 0.926 (*p* = 0.0042), and for D_2_′ from 0.822 to 0.856 (*p* = 0.074). For ROIs excluding centrally deviating areas, these improvements were observed to a lesser degree. For example, by using the 10th percentile instead of mean value, AUC values could only be improved for ADC from 0.958 to 0.975 (*p* = 0.13) and for D_1_′ from 0.902 to 0.935 (*p* = 0.038) and not for D_2_′. The additional comparison using ROIs excluding centrally deviating areas in case of mean value analysis and including such areas in case of histogram analysis, no significant differences were found for ADC, D_1_′ and D_2_′. This means, that the use of low percentiles can replace the elaborate exclusion of centrally deviating areas by hand without reducing the diagnostic accuracy. At 3.0 T, where there were fewer cases with centrally deviating areas, similar results were obtained but with higher *p*-values.

At both field strengths, the 5th and 10th percentiles of D*′ lead to significantly higher AUC values than the mean values, regardless of whether centrally deviating areas were included or excluded or excluded only in case of mean value analysis. For example, by using the 5th percentile instead of the mean value, AUC values could be improved from 0.515 to 0.646 (*p* = 0.00085) at 1.5 T and from 0.559 to 0.717 (*p* = 0.0079) at 3.0 T for ROIs excluding centrally deviating areas. This behavior also tended to be observed for f_1_′. For example, by using the 5th percentile instead of the mean value, AUC values could be improved from 0.622 to 0.708 (*p* = 0.034) at 1.5 T and from 0.661 to 0.681 (*p* = 0.74) at 3.0 T for ROIs excluding centrally deviating areas. All other histogram metrics including skewness and kurtosis performed with lower or not significantly different AUC values compared to the ROI mean values.

## Discussion

The main findings of the present study were: (1) No significant differences in diagnostic performance were found between 2D- and 3D-ROIs even if only slices with good image quality were included. (2) Differentiation was more accurate when centrally deviating areas were excluded from ROIs. (3) When such areas were included, diagnostic accuracy of diffusion sensitive parameters was improved by histogram analysis of the ROIs using low percentiles instead of mean values. (4) Diagnostic accuracy of perfusion parameters, especially of D*′ was improved by histogram analysis using low percentiles instead of mean values, regardless of whether centrally deviating areas were in- or excluded.

To our knowledge, to date no systematic evaluation of different ROI placement and analysis methods for liver lesion analysis by IVIM-derived DWI parameters has been performed. However, it is important for potential clinical use of IVIM DWI techniques for lesion characterization to establish an appropriate ROI placement and analysis strategy as simple as possible that leads to highest possible diagnostic accuracy.

The technically simplest way for ROI placement in clinical practice is to draw a single 2D-ROI on a representative slice encompassing the whole lesion including centrally deviating areas. In scientific studies, however, 3D-volume ROIs are often used e.g. together with automated segmentation software. In the present work we performed comparisons with respect to ROI-type (2D on a reference slice, 3DA for whole-tumor volume, 3DG considering only “good” slices) and tumor tissue by inclusion and exclusion of centrally deviating areas. For different ROI-types, we did not find significant differences in diagnostic accuracy of ADC and IVIM parameters. Compared to 3D-whole-lesion ROIs (3DA), the inclusion of only “good” slices (3DG) or the selection of a ROI on a reference slice (2D) was expected to improve diagnostic accuracy due to less influence of artifacts, pixel misalignments and partial volume effects. However, this effect was hard to find. One reason might be that in case of whole-tumor 3DA volumes negative influences by “bad” slices were compensated by improved statistics due to higher number of included voxels compared to 3DG and 2D. More voxel averaging and thus a better noise robustness was noticeable especially in small lesions (see Table S3). A previous study on prostate cancer also yielded no improved diagnostic performance using 3D-ROIs instead of 2D-ROIs^35^. Although further studies on a larger population with liver lesions are needed to confirm the finding of this study, the analysis of a central representative slice of “good” image quality seems to be sufficient for reliable lesion discrimination and is applicable in clinical practice and less time consuming.

The exclusion of centrally deviating areas significantly improves the diagnostic accuracy of diffusion parameters, as was to be expected. For perfusion parameters no differences were found. A previous study on breast lesions, also found improved accuracy of differential diagnosis for ADC in ROIs including only viable tissue instead of whole tumor^29^. Necrosis, cystic areas and scars increase the diffusion coefficient of a lesion at random due to the admixture of varied proportions of high values. Especially in case of necrosis, the malignancy of tumors may be masked by measurement of a higher ADC due to varying amounts of necrotic tissue. Perfusion parameters, in contrast, are low in necrosis which further reduces the already small values in malignant tumors. In liver metastases, a correlation was found between diffusion parameters and liver tumor necrosis, but not for perfusion parameters^36^.

For lesion assessment, the exclusion of centrally deviating areas is more time consuming and, therefore, not a routine clinical practice and can be challenging for unexperienced radiologists. Thus, automated segmentation would be helpful. In this respect, histogram analysis can provide additional quantitative metrics beyond the mean value of a ROI, which reflect the heterogeneity of pathologic changes without additional imaging^7^. In our study, histogram analysis of ROIs including centrally deviating areas showed that low percentiles led to similar diagnostic accuracy for ADC and diffusion coefficients than mean value analysis of ROIs without such areas. Thus, this method may be of use to automatically determine voxels of viable tumor for ADC and IVIM analysis. In some other studies, it was also shown that diagnostic accuracy of ADC and D in whole-lesion ROI analysis was improved when low percentiles were used instead of mean values, e.g. in predicting microvascular invasion of hepatocellular carcinoma^37^, differentiation of malignancy in breast and testicular lesions^31,38^, differentiating of different grades of prostate cancer^39^, and gliomas^40–42^.

Furthermore, of special interest is the finding that for the perfusion parameters, especially D*, diagnostic accuracy in lesion discrimination was significantly improved by the use of low percentiles instead of mean values regardless of whether centrally deviating areas were included or excluded or excluded only in case of mean value analysis. Because D* depends on blood flow velocity and length of microvessel segments^3,4^, this may indicate that differences between benign and malignant lesions exist especially for small vessels. Other studies investigating histogram analysis for IVIM perfusion parameters in liver lesions are rare. There is one other study investigating hepatocellular carcinoma with and without microvascular invasion, but no significant differences were found for parameters D* and f, neither for mean values nor for low percentiles^37^.

This study has several limitations. First, it was a retrospective study with inherent methodological limitations. For example, due to the lack of raw data, no motion correction of the individual images^43^ could be performed before averaging. Second, although the total number of lesions included was relatively large, only common lesion types were analyzed, which may affect the generalizability of the results. Also, there was a relatively small number of patients who underwent MRI examination at 3.0 T MRI system and, therefore, statistical power was lower compared to 1.5 T. We included a typical clinical patient cohort of a large tertiary reference center so that not only large lesions were included. Therefore, a study including more large lesions may show differences between 2D- and 3D-volume measurements. On the other hand, not even tendencies concerning differences of 2D- and 3D-ROIs were found in the present study.

In conclusion, using representative 2D-ROIs seems to be sufficient for reliable liver lesion discrimination in routine clinical practice. Central necrosis, cystic components or scars should be excluded from ROIs either by hand or by computing low percentiles of diffusion coefficients instead of mean values.

## Supplementary Information

Supplementary Tables.

## Data Availability

The datasets generated during and/or analyzed during the current study are available from the corresponding author on reasonable request.

## References

[1] Shenoy-Bhangle A, Baliyan V, Kordbacheh H, Guimaraes AR, Kambadakone A (2017). Diffusion weighted magnetic resonance imaging of liver: Principles, clinical applications and recent updates. World J. Hepatol..

[2] Thoeny HC, Ross BD (2010). Predicting and monitoring cancer treatment response with diffusion-weighted MRI. J. Magn. Reson. Imaging.

[3] Le Bihan D (1988). Separation of diffusion and perfusion in intravoxel incoherent motion MR imaging. Radiology.

[4] Koh DM, Collins DJ, Orton MR (2011). Intravoxel incoherent motion in body diffusion-weighted MRI: Reality and challenges. Am. J. Roentgenol..

[5] Koh DM, Collins DJ (2007). Diffusion-weighted MRI in the body: Applications and challenges in oncology. Am. J. Roentgenol..

[6] Koh DM (2014). Science to practice: Can intravoxel incoherent motion diffusion-weighted MR imaging be used to assess tumor response to antivascular drugs?. Radiology.

[7] Padhani AR (2009). Diffusion-weighted magnetic resonance imaging as a cancer biomarker: Consensus and recommendations. Neoplasia.

[8] Meyer H-J (2021). Associations between IVIM histogram parameters and histopathology in rectal cancer. Magn. Reson. Imaging.

[9] Lee HJ (2014). Tumor perfusion-related parameter of diffusion-weighted magnetic resonance imaging: Correlation with histological microvessel density. Magn. Reson. Med..

[10] Kim S (2012). Interstitial fluid pressure correlates with intravoxel incoherent motion imaging metrics in a mouse mammary carcinoma model. NMR Biomed..

[11] Lee Y (2015). Intravoxel incoherent motion diffusion-weighted MR imaging of the liver: Effect of triggering methods on regional variability and measurement repeatability of quantitative parameters. Radiology.

[12] Cho GY (2012). A versatile flow phantom for intravoxel incoherent motion MRI. Magn. Reson..

[13] Kakite S (2015). Hepatocellular carcinoma: Short-term reproducibility of apparent diffusion coefficient and intravoxel incoherent motion parameters at 3.0T. J Magn Reson Imaging.

[14] Andreou A (2013). Measurement reproducibility of perfusion fraction and pseudodiffusion coefficient derived by intravoxel incoherent motion diffusion-weighted MR imaging in normal liver and metastases. Eur Radiol.

[15] Wang M (2016). Evaluation of hepatic tumors using intravoxel incoherent motion diffusion-weighted MRI. Med. Sci. Monit..

[16] Colagrande S (2013). Focal liver lesion classification and characterization in noncirrhotic liver: A prospective comparison of diffusion-weighted magnetic resonance-related parameters. J. Comput. Assist. Tomogr..

[17] Doblas S (2013). Determination of malignancy and characterization of hepatic tumor type with diffusion-weighted magnetic resonance imaging: Comparison of apparent diffusion coefficient and intravoxel incoherent motion-derived measurements. Invest. Radiol..

[18] Ichikawa S (2013). Intravoxel incoherent motion imaging of focal hepatic lesions. J. Magn. Reson. Imaging.

[19] Luo M, Zhang L, Jiang X, Zhang W (2017). Intravoxel incoherent motion diffusion-weighted imaging: Evaluation of the differentiation of solid hepatic lesions. Transl. Oncol..

[20] Coenegrachts K (2009). Evaluation of true diffusion, perfusion factor, and apparent diffusion coefficient in non-necrotic liver metastases and uncomplicated liver hemangiomas using black-blood echo planar imaging. Eur. J. Radiol..

[21] Mürtz P (2018). Accurate IVIM model-based liver lesion characterisation can be achieved with only three b-value DWI. Eur. Radiol..

[22] Mürtz P (2016). Intravoxel incoherent motion model-based analysis of diffusion-weighted magnetic resonance imaging with 3 b-values for response assessment in locoregional therapy of hepatocellular carcinoma. OncoTargets Ther..

[23] Mürtz P (2019). Is liver lesion characterisation by simplified IVIM DWI also feasible at 3.0 T?. Eur. Radiol..

[24] Pieper CC (2016). Incidence and risk factors of early arterial blood flow stasis during first radioembolization of primary and secondary liver malignancy using resin microspheres: An initial single-center analysis. Eur. Radiol..

[25] Pieper CC (2016). Evaluation of a simplified intravoxel incoherent motion (IVIM) analysis of diffusion-weighted imaging for prediction of tumor size changes and imaging response in breast cancer liver metastases undergoing radioembolization: A retrospective single center analysis. Medicine (Baltimore).

[26] Pieper C (2016). The value of intravoxel incoherent motion model-based diffusion-weighted imaging for outcome prediction in resin-based radioembolization of breast cancer liver metastases. OncoTargets Ther..

[27] Pieper CC, Sprinkart AM, Kukuk GM, Mürtz P (2019). Short-term measurement repeatability of a simplified intravoxel incoherent motion (IVIM) analysis for routine clinical diffusion-weighted imaging in malignant liver lesions and liver parenchyma at 1.5 T. RöFo Fortschritte Auf Dem Geb. Röntgenstrahlen Bildgeb. Verfahr..

[28] Ma C (2017). Effect of region of interest size on ADC measurements in pancreatic adenocarcinoma. Cancer Imaging.

[29] Gity M, Moradi B, Arami R, Arabkheradmand A, Kazemi MA (2018). Two different methods of region-of-interest placement for differentiation of benign and malignant breast lesions by apparent diffusion coefficient value. Asian Pac. J. Cancer Prev..

[30] Bickel H (2017). Diffusion-weighted imaging of breast lesions: Region-of-interest placement and different ADC parameters influence apparent diffusion coefficient values. Eur. Radiol..

[31] Suo S (2016). Characterization of breast masses as benign or malignant at 3.0T MRI with whole-lesion histogram analysis of the apparent diffusion coefficient. J. Magn. Reson. Imaging.

[32] Bruix J, Sherman M (2011). Management of hepatocellular carcinoma: An update. Hepatology.

[33] Robin X (2011). pROC: An open-source package for R and S+ to analyze and compare ROC curves. BMC Bioinformatics.

[34] DeLong ER, DeLong DM, Clarke-Pearson DL (1988). Comparing the areas under two or more correlated receiver operating characteristic curves: A nonparametric approach. Biometrics.

[35] Tamada T (2018). Apparent diffusion coefficient values of prostate cancer: Comparison of 2D and 3D ROIs. Am. J. Roentgenol..

[36] Chiaradia M (2014). Intravoxel incoherent motion (IVIM) MR imaging of colorectal liver metastases: Are we only looking at tumor necrosis?. J Magn Reson Imaging.

[37] Li H (2018). Preoperative histogram analysis of intravoxel incoherent motion (IVIM) for predicting microvascular invasion in patients with single hepatocellular carcinoma. Eur. J. Radiol..

[38] Fan C (2020). Discrimination between benign and malignant testicular lesions using volumetric apparent diffusion coefficient histogram analysis. Eur. J. Radiol..

[39] Donati OF (2014). Prostate cancer aggressiveness: Assessment with whole-lesion histogram analysis of the apparent diffusion coefficient. Radiology.

[40] Horvath-Rizea D (2018). The value of whole lesion ADC histogram profiling to differentiate between morphologically indistinguishable ring enhancing lesions-comparison of glioblastomas and brain abscesses. Oncotarget.

[41] Lu SS (2015). Histogram analysis of apparent diffusion coefficient maps for differentiating primary CNS lymphomas from tumefactive demyelinating lesions. Am. J. Roentgenol..

[42] Kang Y (2011). Gliomas: Histogram analysis of apparent diffusion coefficient maps with standard- or high-b-value diffusion-weighted MR imaging—Correlation with tumor grade. Radiology.

[43] Pathak R (2019). Considering tumour volume for motion corrected DWI of colorectal liver metastases increases sensitivity of ADC to detect treatment-induced changes. Sci. Rep..

