# Supplementary material for: Comparison of different ROI analysis methods for liver lesion characterization with simplified intravoxel incoherent motion (IVIM)
Source: Sci Rep. 2021 Nov 23;11:22752. doi: 10.1038/s41598-021-01108-6 (PMC8610969; doi:10.1038/s41598-021-01108-6)
Supplement: Supplementary file 1 — Supplementary Tables. [file 41598_2021_1108_MOESM1_ESM.docx]

**Supplementary table 1.** Results of parameter values obtained by histogram analysis of the 2D ROIs and receiver operating characteristic (ROC) analysis of benign and malignant liver lesions (see Table 1) at 1.5 T (a) and 3.0 T (b).

a) 1.5 T

| **Par** | | **Malignant** | | | **Benign** | | | **Dir** | **AUC** | **CI1** | **CI2** | **Cut-off** | **Sen** | **Spec** | **Acc** |
| --- | --- | --- | --- | --- | --- | --- | --- | --- | --- | --- | --- | --- | --- | --- | --- |
|  |  | **V** | **SD** | **N** | **V** | **SD** | **N** |  |  |  |  |  |  |  |  |
| **ROIs including necrosis, cystic components, scars** | | | | | | | | | | | | | | | |
| **ADC** | Mean | 1182 | 216 | 74 | 1712 | 329 | 35 | > | 0.925 | 0.878 | 0.972 | 1335.8 | 0.797 | 0.914 | 0.835 |
|  | Median | 1160 | 221 | 74 | 1697 | 332 | 35 | > | 0.928 | 0.881 | 0.974 | 1318.5 | 0.824 | 0.914 | 0.853 |
|  | Std | 243 | 117 | 74 | 227 | 139 | 35 | < | 0.597 | 0.477 | 0.718 | 179.3 | 0.676 | 0.543 | 0.633 |
|  | 5^th^ Perc | 823 | 189 | 74 | 1367 | 232 | 35 | > | 0.971 | 0.945 | 0.997 | 1102.1 | 0.932 | 0.914 | 0.927 |
|  | 10^th^ Perc | 894 | 180 | 74 | 1445 | 247 | 35 | > | 0.969 | 0.942 | 0.996 | 1188.8 | 0.959 | 0.886 | 0.936 |
|  | 25^th^ Perc | 1013 | 189 | 74 | 1568 | 286 | 35 | > | 0.956 | 0.921 | 0.991 | 1245.5 | 0.892 | 0.914 | 0.899 |
|  | 75^th^ Perc | 1337 | 287 | 74 | 1859 | 431 | 35 | > | 0.874 | 0.808 | 0.940 | 1414.9 | 0.716 | 0.914 | 0.780 |
|  | 90^th^ Perc | 1505 | 329 | 74 | 1999 | 480 | 35 | > | 0.831 | 0.753 | 0.908 | 1653.7 | 0.797 | 0.714 | 0.771 |
|  | 95^th^ Perc | 1607 | 356 | 74 | 2087 | 483 | 35 | > | 0.815 | 0.735 | 0.896 | 1787.4 | 0.784 | 0.714 | 0.761 |
|  | Skewness | 0.5 | 0.6 | 74 | 0.3 | 0.9 | 35 | < | 0.566 | 0.444 | 0.689 | 0.5 | 0.459 | 0.714 | 0.541 |
|  | Kurtosis | 3.8 | 1.7 | 74 | 4.5 | 2.3 | 35 | > | 0.594 | 0.475 | 0.713 | 4.1 | 0.743 | 0.514 | 0.670 |
| **D_1_'** | Mean | 1115 | 224 | 74 | 1600 | 401 | 35 | > | 0.866 | 0.796 | 0.935 | 1130.4 | 0.622 | 0.943 | 0.725 |
|  | Median | 1090 | 230 | 74 | 1586 | 398 | 35 | > | 0.872 | 0.805 | 0.939 | 1125.5 | 0.635 | 0.943 | 0.734 |
|  | Std | 225 | 119 | 74 | 197 | 127 | 35 | < | 0.614 | 0.494 | 0.733 | 134.6 | 0.824 | 0.429 | 0.697 |
|  | 5^th^ Perc | 788 | 186 | 74 | 1299 | 307 | 35 | > | 0.927 | 0.879 | 0.974 | 1007.8 | 0.851 | 0.829 | 0.844 |
|  | 10^th^ Perc | 851 | 183 | 74 | 1370 | 329 | 35 | > | 0.926 | 0.878 | 0.974 | 1048.1 | 0.865 | 0.829 | 0.853 |
|  | 25^th^ Perc | 955 | 194 | 74 | 1480 | 360 | 35 | > | 0.910 | 0.856 | 0.963 | 1072.0 | 0.703 | 0.943 | 0.780 |
|  | 75^th^ Perc | 1260 | 298 | 74 | 1726 | 484 | 35 | > | 0.807 | 0.721 | 0.892 | 1360.7 | 0.743 | 0.743 | 0.743 |
|  | 90^th^ Perc | 1421 | 339 | 74 | 1847 | 529 | 35 | > | 0.754 | 0.656 | 0.853 | 1818.7 | 0.905 | 0.571 | 0.798 |
|  | 95^th^ Perc | 1518 | 367 | 74 | 1920 | 544 | 35 | > | 0.736 | 0.633 | 0.838 | 1868.4 | 0.892 | 0.571 | 0.789 |
|  | Skewness | 0.4 | 0.6 | 74 | 0.1 | 1.0 | 35 | < | 0.667 | 0.540 | 0.795 | 0.1 | 0.716 | 0.657 | 0.697 |
|  | Kurtosis | 3.5 | 1.5 | 74 | 4.6 | 2.6 | 35 | > | 0.629 | 0.516 | 0.743 | 4.3 | 0.824 | 0.400 | 0.688 |
| **D_2_'** | Mean | 990 | 280 | 74 | 1442 | 433 | 35 | > | 0.822 | 0.733 | 0.911 | 1105.0 | 0.689 | 0.857 | 0.743 |
|  | Median | 967 | 283 | 74 | 1435 | 428 | 35 | > | 0.832 | 0.745 | 0.919 | 1124.5 | 0.743 | 0.829 | 0.771 |
|  | Std | 236 | 116 | 74 | 216 | 132 | 35 | < | 0.591 | 0.470 | 0.712 | 131.8 | 0.892 | 0.314 | 0.706 |
|  | 5^th^ Perc | 639 | 254 | 74 | 1098 | 411 | 35 | > | 0.841 | 0.746 | 0.935 | 924.1 | 0.865 | 0.743 | 0.826 |
|  | 10^th^ Perc | 712 | 254 | 74 | 1190 | 403 | 35 | > | 0.856 | 0.772 | 0.940 | 975.7 | 0.878 | 0.771 | 0.844 |
|  | 25^th^ Perc | 826 | 264 | 74 | 1300 | 409 | 35 | > | 0.852 | 0.768 | 0.937 | 1066.7 | 0.851 | 0.743 | 0.817 |
|  | 75^th^ Perc | 1141 | 331 | 74 | 1582 | 492 | 35 | > | 0.792 | 0.700 | 0.885 | 1275.7 | 0.730 | 0.800 | 0.752 |
|  | 90^th^ Perc | 1303 | 378 | 74 | 1718 | 540 | 35 | > | 0.753 | 0.655 | 0.852 | 1439.1 | 0.730 | 0.743 | 0.734 |
|  | 95^th^ Perc | 1405 | 409 | 74 | 1792 | 561 | 35 | > | 0.720 | 0.619 | 0.822 | 1505.8 | 0.689 | 0.686 | 0.688 |
|  | Skewness | 0.3 | 0.6 | 74 | 0.1 | 0.9 | 35 | < | 0.620 | 0.495 | 0.746 | 0.1 | 0.622 | 0.629 | 0.624 |
|  | Kurtosis | 3.5 | 1.4 | 74 | 4.1 | 2.9 | 35 | > | 0.571 | 0.453 | 0.690 | 3.5 | 0.689 | 0.543 | 0.642 |
| **f_1_'** | Mean | 64 | 31 | 74 | 97 | 70 | 35 | > | 0.621 | 0.490 | 0.753 | 110.7 | 0.905 | 0.457 | 0.761 |
|  | Median | 50 | 37 | 74 | 84 | 74 | 35 | > | 0.620 | 0.490 | 0.751 | 109.2 | 0.932 | 0.429 | 0.771 |
|  | Std | 58 | 20 | 74 | 60 | 26 | 35 | < | 0.507 | 0.384 | 0.629 | 41.1 | 0.824 | 0.286 | 0.651 |
|  | 5^th^ Perc | 2 | 8 | 74 | 28 | 44 | 35 | > | 0.712 | 0.616 | 0.807 | 10.3 | 0.946 | 0.457 | 0.789 |
|  | 10^th^ Perc | 6 | 13 | 74 | 36 | 50 | 35 | > | 0.671 | 0.561 | 0.781 | 28.2 | 0.932 | 0.486 | 0.789 |
|  | 25^th^ Perc | 22 | 26 | 74 | 54 | 62 | 35 | > | 0.637 | 0.515 | 0.760 | 75.8 | 0.973 | 0.400 | 0.789 |
|  | 75^th^ Perc | 91 | 43 | 74 | 124 | 86 | 35 | > | 0.606 | 0.474 | 0.738 | 149.0 | 0.905 | 0.429 | 0.752 |
|  | 90^th^ Perc | 136 | 50 | 74 | 178 | 96 | 35 | > | 0.612 | 0.484 | 0.741 | 181.6 | 0.824 | 0.486 | 0.716 |
|  | 95^th^ Perc | 171 | 61 | 74 | 211 | 103 | 35 | > | 0.594 | 0.468 | 0.720 | 199.9 | 0.716 | 0.543 | 0.661 |
|  | Skewness | 1.4 | 1.1 | 74 | 1.3 | 0.9 | 35 | < | 0.539 | 0.423 | 0.654 | 1.0 | 0.595 | 0.543 | 0.578 |
|  | Kurtosis | 7.0 | 5.7 | 74 | 6.3 | 4.6 | 35 | < | 0.531 | 0.414 | 0.647 | 6.5 | 0.419 | 0.743 | 0.523 |
| **f_2_'** | Mean | 145 | 96 | 74 | 191 | 104 | 35 | > | 0.656 | 0.546 | 0.766 | 198.9 | 0.838 | 0.429 | 0.706 |
|  | Median | 135 | 107 | 74 | 180 | 114 | 35 | > | 0.637 | 0.524 | 0.749 | 195.2 | 0.838 | 0.429 | 0.706 |
|  | Std | 82 | 27 | 74 | 90 | 34 | 35 | > | 0.534 | 0.409 | 0.659 | 106.0 | 0.865 | 0.371 | 0.706 |
|  | 5^th^ Perc | 34 | 69 | 74 | 65 | 85 | 35 | > | 0.637 | 0.528 | 0.746 | 12.8 | 0.676 | 0.657 | 0.670 |
|  | 10^th^ Perc | 50 | 77 | 74 | 88 | 95 | 35 | > | 0.639 | 0.526 | 0.753 | 38.6 | 0.635 | 0.657 | 0.642 |
|  | 25^th^ Perc | 87 | 94 | 74 | 128 | 107 | 35 | > | 0.633 | 0.518 | 0.748 | 74.9 | 0.595 | 0.714 | 0.633 |
|  | 75^th^ Perc | 190 | 112 | 74 | 244 | 120 | 35 | > | 0.651 | 0.538 | 0.763 | 236.7 | 0.770 | 0.514 | 0.688 |
|  | 90^th^ Perc | 250 | 110 | 74 | 313 | 124 | 35 | > | 0.654 | 0.543 | 0.766 | 267.1 | 0.676 | 0.600 | 0.651 |
|  | 95^th^ Perc | 292 | 112 | 74 | 355 | 124 | 35 | > | 0.642 | 0.529 | 0.755 | 302.2 | 0.608 | 0.686 | 0.633 |
|  | Skewness | 0.8 | 1.0 | 74 | 0.6 | 0.8 | 35 | < | 0.564 | 0.448 | 0.680 | 0.2 | 0.784 | 0.400 | 0.661 |
|  | Kurtosis | 4.7 | 2.8 | 74 | 4.1 | 2.2 | 35 | < | 0.553 | 0.439 | 0.667 | 4.6 | 0.392 | 0.800 | 0.523 |
| **D*'** | Mean | 18370 | 8332 | 74 | 21200 | 13245 | 35 | > | 0.529 | 0.401 | 0.656 | 25008.0 | 0.811 | 0.371 | 0.670 |
|  | Median | 15221 | 7583 | 74 | 18128 | 11797 | 35 | > | 0.545 | 0.418 | 0.672 | 23471.1 | 0.878 | 0.343 | 0.706 |
|  | Std | 13407 | 5329 | 74 | 12537 | 7280 | 35 | < | 0.547 | 0.418 | 0.677 | 8147.4 | 0.824 | 0.400 | 0.688 |
|  | 5^th^ Perc | 4244 | 3413 | 74 | 7551 | 6507 | 35 | > | 0.658 | 0.542 | 0.773 | 7302.3 | 0.878 | 0.400 | 0.725 |
|  | 10^th^ Perc | 5978 | 3918 | 74 | 9156 | 7214 | 35 | > | 0.617 | 0.496 | 0.738 | 8297.7 | 0.824 | 0.429 | 0.697 |
|  | 25^th^ Perc | 9787 | 5414 | 74 | 12711 | 9180 | 35 | > | 0.558 | 0.430 | 0.686 | 18440.6 | 0.946 | 0.257 | 0.725 |
|  | 75^th^ Perc | 23068 | 11106 | 74 | 26367 | 16092 | 35 | > | 0.532 | 0.407 | 0.657 | 33961.5 | 0.865 | 0.314 | 0.688 |
|  | 90^th^ Perc | 34049 | 15321 | 74 | 37516 | 22455 | 35 | > | 0.515 | 0.387 | 0.644 | 58602.8 | 0.959 | 0.257 | 0.734 |
|  | 95^th^ Perc | 44146 | 19072 | 74 | 44682 | 26415 | 35 | < | 0.519 | 0.391 | 0.648 | 26554.9 | 0.797 | 0.343 | 0.651 |
|  | Skewness | 2.2 | 1.2 | 74 | 1.6 | 1.1 | 35 | < | 0.679 | 0.562 | 0.796 | 1.7 | 0.784 | 0.657 | 0.743 |
|  | Kurtosis | 12.6 | 11.8 | 74 | 8.3 | 8.1 | 35 | < | 0.686 | 0.570 | 0.803 | 7.8 | 0.703 | 0.743 | 0.716 |
| **ROIs excluding necrosis, cystic components, scars** | | | | | | | | | | | | | | | |
| **ADC** | Mean | 1124 | 180 | 74 | 1692 | 313 | 35 | > | 0.958 | 0.922 | 0.993 | 1338.5 | 0.892 | 0.914 | 0.899 |
|  | Median | 1109 | 188 | 74 | 1687 | 329 | 35 | > | 0.955 | 0.918 | 0.993 | 1322.8 | 0.878 | 0.914 | 0.890 |
|  | Std | 208 | 78 | 74 | 209 | 108 | 35 | < | 0.543 | 0.420 | 0.667 | 178.9 | 0.622 | 0.543 | 0.596 |
|  | 5^th^ Perc | 807 | 196 | 74 | 1365 | 231 | 35 | > | 0.974 | 0.949 | 0.999 | 1099.6 | 0.946 | 0.914 | 0.936 |
|  | 10^th^ Perc | 875 | 183 | 74 | 1442 | 247 | 35 | > | 0.975 | 0.950 | 0.999 | 1187.3 | 0.973 | 0.886 | 0.945 |
|  | 25^th^ Perc | 986 | 180 | 74 | 1565 | 287 | 35 | > | 0.968 | 0.939 | 0.997 | 1239.2 | 0.932 | 0.914 | 0.927 |
|  | 75^th^ Perc | 1247 | 198 | 74 | 1819 | 377 | 35 | > | 0.933 | 0.885 | 0.981 | 1414.4 | 0.824 | 0.914 | 0.853 |
|  | 90^th^ Perc | 1395 | 222 | 74 | 1948 | 412 | 35 | > | 0.904 | 0.844 | 0.964 | 1593.1 | 0.851 | 0.800 | 0.835 |
|  | 95^th^ Perc | 1487 | 245 | 74 | 2039 | 416 | 35 | > | 0.894 | 0.830 | 0.957 | 1617.7 | 0.743 | 0.886 | 0.789 |
|  | Skewness | 0.4 | 0.6 | 74 | 0.3 | 0.9 | 35 | < | 0.553 | 0.429 | 0.676 | 0.0 | 0.770 | 0.371 | 0.642 |
|  | Kurtosis | 3.8 | 1.5 | 74 | 4.6 | 2.2 | 35 | > | 0.610 | 0.490 | 0.729 | 5.0 | 0.865 | 0.400 | 0.716 |
| **D_1_'** | Mean | 1057 | 188 | 74 | 1580 | 387 | 35 | > | 0.902 | 0.842 | 0.962 | 1173.6 | 0.757 | 0.886 | 0.798 |
|  | Median | 1041 | 197 | 74 | 1576 | 395 | 35 | > | 0.905 | 0.847 | 0.963 | 1128.1 | 0.730 | 0.943 | 0.798 |
|  | Std | 186 | 75 | 74 | 178 | 85 | 35 | < | 0.553 | 0.426 | 0.679 | 118.1 | 0.838 | 0.371 | 0.688 |
|  | 5^th^ Perc | 776 | 185 | 74 | 1296 | 307 | 35 | > | 0.934 | 0.889 | 0.979 | 916.2 | 0.784 | 0.914 | 0.826 |
|  | 10^th^ Perc | 836 | 184 | 74 | 1368 | 328 | 35 | > | 0.935 | 0.891 | 0.978 | 967.6 | 0.757 | 0.943 | 0.817 |
|  | 25^th^ Perc | 932 | 187 | 74 | 1477 | 360 | 35 | > | 0.929 | 0.882 | 0.976 | 1078.6 | 0.811 | 0.943 | 0.853 |
|  | 75^th^ Perc | 1170 | 209 | 74 | 1689 | 437 | 35 | > | 0.865 | 0.792 | 0.939 | 1360.7 | 0.851 | 0.743 | 0.817 |
|  | 90^th^ Perc | 1305 | 231 | 74 | 1791 | 461 | 35 | > | 0.832 | 0.747 | 0.918 | 1818.7 | 0.986 | 0.571 | 0.853 |
|  | 95^th^ Perc | 1387 | 254 | 74 | 1862 | 470 | 35 | > | 0.811 | 0.719 | 0.903 | 1643.2 | 0.878 | 0.657 | 0.807 |
|  | Skewness | 0.4 | 0.6 | 74 | 0.1 | 1.0 | 35 | < | 0.648 | 0.518 | 0.778 | 0.1 | 0.716 | 0.657 | 0.697 |
|  | Kurtosis | 3.7 | 1.4 | 74 | 4.8 | 2.6 | 35 | > | 0.627 | 0.509 | 0.744 | 4.8 | 0.878 | 0.400 | 0.725 |
| **D_2_'** | Mean | 939 | 250 | 74 | 1423 | 416 | 35 | > | 0.864 | 0.783 | 0.946 | 1142.5 | 0.838 | 0.829 | 0.835 |
|  | Median | 928 | 256 | 74 | 1427 | 424 | 35 | > | 0.862 | 0.779 | 0.944 | 1124.5 | 0.824 | 0.829 | 0.826 |
|  | Std | 195 | 68 | 74 | 197 | 98 | 35 | < | 0.527 | 0.399 | 0.656 | 131.8 | 0.851 | 0.343 | 0.688 |
|  | 5^th^ Perc | 633 | 251 | 74 | 1094 | 410 | 35 | > | 0.844 | 0.750 | 0.938 | 923.4 | 0.878 | 0.743 | 0.835 |
|  | 10^th^ Perc | 704 | 250 | 74 | 1186 | 402 | 35 | > | 0.861 | 0.778 | 0.945 | 975.0 | 0.878 | 0.771 | 0.844 |
|  | 25^th^ Perc | 811 | 255 | 74 | 1297 | 408 | 35 | > | 0.863 | 0.781 | 0.945 | 1027.7 | 0.838 | 0.771 | 0.817 |
|  | 75^th^ Perc | 1059 | 258 | 74 | 1547 | 448 | 35 | > | 0.851 | 0.767 | 0.934 | 1242.5 | 0.824 | 0.829 | 0.826 |
|  | 90^th^ Perc | 1190 | 277 | 74 | 1661 | 462 | 35 | > | 0.830 | 0.739 | 0.920 | 1439.1 | 0.878 | 0.743 | 0.835 |
|  | 95^th^ Perc | 1272 | 293 | 74 | 1728 | 467 | 35 | > | 0.807 | 0.714 | 0.901 | 1555.0 | 0.905 | 0.629 | 0.817 |
|  | Skewness | 0.2 | 0.6 | 74 | 0.0 | 0.9 | 35 | < | 0.595 | 0.472 | 0.717 | 0.0 | 0.608 | 0.600 | 0.606 |
|  | Kurtosis | 3.6 | 1.2 | 74 | 4.3 | 2.9 | 35 | > | 0.557 | 0.435 | 0.678 | 3.8 | 0.662 | 0.514 | 0.615 |
| **f_1_'** | Mean | 63 | 31 | 74 | 97 | 70 | 35 | > | 0.622 | 0.491 | 0.754 | 114.5 | 0.932 | 0.457 | 0.780 |
|  | Median | 50 | 36 | 74 | 83 | 74 | 35 | > | 0.615 | 0.484 | 0.747 | 107.6 | 0.919 | 0.429 | 0.761 |
|  | Std | 57 | 21 | 74 | 60 | 26 | 35 | > | 0.512 | 0.391 | 0.634 | 68.0 | 0.784 | 0.371 | 0.651 |
|  | 5^th^ Perc | 2 | 8 | 74 | 28 | 44 | 35 | > | 0.708 | 0.611 | 0.805 | 10.8 | 0.946 | 0.457 | 0.789 |
|  | 10^th^ Perc | 6 | 13 | 74 | 37 | 50 | 35 | > | 0.675 | 0.566 | 0.783 | 29.3 | 0.932 | 0.486 | 0.789 |
|  | 25^th^ Perc | 21 | 26 | 74 | 54 | 62 | 35 | > | 0.638 | 0.516 | 0.760 | 75.8 | 0.973 | 0.400 | 0.789 |
|  | 75^th^ Perc | 91 | 43 | 74 | 125 | 87 | 35 | > | 0.603 | 0.471 | 0.735 | 149.0 | 0.892 | 0.429 | 0.743 |
|  | 90^th^ Perc | 136 | 54 | 74 | 178 | 97 | 35 | > | 0.612 | 0.485 | 0.739 | 181.6 | 0.811 | 0.486 | 0.706 |
|  | 95^th^ Perc | 170 | 67 | 74 | 211 | 103 | 35 | > | 0.604 | 0.480 | 0.729 | 199.9 | 0.730 | 0.543 | 0.670 |
|  | Skewness | 1.3 | 1.0 | 74 | 1.3 | 0.9 | 35 | < | 0.507 | 0.391 | 0.623 | 1.1 | 0.527 | 0.571 | 0.541 |
|  | Kurtosis | 6.2 | 4.7 | 74 | 6.2 | 4.4 | 35 | > | 0.514 | 0.396 | 0.632 | 4.2 | 0.500 | 0.600 | 0.532 |
| **f_2_'** | Mean | 141 | 96 | 74 | 191 | 104 | 35 | > | 0.672 | 0.563 | 0.781 | 140.0 | 0.622 | 0.657 | 0.633 |
|  | Median | 130 | 107 | 74 | 180 | 114 | 35 | > | 0.660 | 0.549 | 0.771 | 115.0 | 0.541 | 0.771 | 0.615 |
|  | Std | 82 | 29 | 74 | 90 | 34 | 35 | > | 0.553 | 0.432 | 0.674 | 106.2 | 0.865 | 0.371 | 0.706 |
|  | 5^th^ Perc | 32 | 66 | 74 | 65 | 85 | 35 | > | 0.655 | 0.549 | 0.762 | 13.3 | 0.689 | 0.657 | 0.679 |
|  | 10^th^ Perc | 47 | 76 | 74 | 88 | 95 | 35 | > | 0.653 | 0.541 | 0.766 | 47.2 | 0.689 | 0.629 | 0.670 |
|  | 25^th^ Perc | 83 | 93 | 74 | 128 | 107 | 35 | > | 0.649 | 0.535 | 0.762 | 74.9 | 0.581 | 0.714 | 0.624 |
|  | 75^th^ Perc | 186 | 112 | 74 | 244 | 120 | 35 | > | 0.668 | 0.558 | 0.779 | 234.1 | 0.784 | 0.514 | 0.697 |
|  | 90^th^ Perc | 247 | 113 | 74 | 313 | 124 | 35 | > | 0.661 | 0.551 | 0.771 | 243.1 | 0.608 | 0.657 | 0.624 |
|  | 95^th^ Perc | 290 | 118 | 74 | 354 | 124 | 35 | > | 0.643 | 0.532 | 0.754 | 302.8 | 0.635 | 0.686 | 0.651 |
|  | Skewness | 0.8 | 0.9 | 74 | 0.6 | 0.8 | 35 | < | 0.558 | 0.442 | 0.674 | 0.2 | 0.797 | 0.371 | 0.661 |
|  | Kurtosis | 4.5 | 2.8 | 74 | 4.2 | 2.2 | 35 | < | 0.510 | 0.394 | 0.626 | 4.7 | 0.297 | 0.800 | 0.459 |
| **D*'** | Mean | 18837 | 8603 | 74 | 21189 | 13251 | 35 | > | 0.515 | 0.388 | 0.642 | 24996.2 | 0.784 | 0.371 | 0.651 |
|  | Median | 15614 | 7860 | 74 | 18070 | 11799 | 35 | > | 0.534 | 0.408 | 0.661 | 23471.1 | 0.865 | 0.314 | 0.688 |
|  | Std | 13834 | 6019 | 74 | 12613 | 7338 | 35 | < | 0.551 | 0.424 | 0.677 | 8293.7 | 0.797 | 0.400 | 0.670 |
|  | 5^th^ Perc | 4300 | 3473 | 74 | 7497 | 6530 | 35 | > | 0.646 | 0.530 | 0.762 | 7302.3 | 0.878 | 0.400 | 0.725 |
|  | 10^th^ Perc | 6016 | 3972 | 74 | 9115 | 7260 | 35 | > | 0.608 | 0.487 | 0.729 | 12749.1 | 0.959 | 0.286 | 0.743 |
|  | 25^th^ Perc | 9888 | 5441 | 74 | 12653 | 9204 | 35 | > | 0.549 | 0.421 | 0.677 | 18607.5 | 0.946 | 0.257 | 0.725 |
|  | 75^th^ Perc | 23816 | 11288 | 74 | 26310 | 16079 | 35 | > | 0.517 | 0.391 | 0.642 | 34363.1 | 0.878 | 0.314 | 0.697 |
|  | 90^th^ Perc | 34726 | 15571 | 74 | 37576 | 22429 | 35 | > | 0.508 | 0.379 | 0.638 | 57884.9 | 0.959 | 0.257 | 0.734 |
|  | 95^th^ Perc | 45681 | 20922 | 74 | 45049 | 26619 | 35 | < | 0.525 | 0.398 | 0.652 | 26624.7 | 0.797 | 0.343 | 0.651 |
|  | Skewness | 2.1 | 1.0 | 74 | 1.6 | 1.1 | 35 | < | 0.652 | 0.533 | 0.770 | 1.5 | 0.757 | 0.600 | 0.706 |
|  | Kurtosis | 10.7 | 7.3 | 74 | 8.3 | 8.2 | 35 | < | 0.654 | 0.536 | 0.773 | 7.8 | 0.622 | 0.743 | 0.661 |

b) 3.0 T

| **Par** | | **Malignant** | | | **Benign** | | | **Dir** | **AUC** | **CI1** | **CI2** | **Cut-off** | **Sen** | **Spec** | **Acc** |
| --- | --- | --- | --- | --- | --- | --- | --- | --- | --- | --- | --- | --- | --- | --- | --- |
|  |  | **V** | **SD** | **N** | **V** | **SD** | **N** |  |  |  |  |  |  |  |  |
| **ROIs including necrosis, cystic components, scars** | | | | | | | | | | | | | | | |
| **ADC** | Mean | 1120 | 183 | 54 | 1566 | 251 | 19 | > | 0.931 | 0.858 | 1.000 | 1419.4 | 0.963 | 0.789 | 0.918 |
|  | Median | 1110 | 191 | 54 | 1573 | 255 | 19 | > | 0.937 | 0.868 | 1.000 | 1399.9 | 0.963 | 0.789 | 0.918 |
|  | Std | 221 | 83 | 54 | 206 | 82 | 19 | < | 0.578 | 0.425 | 0.731 | 212.7 | 0.593 | 0.684 | 0.616 |
|  | 5^th^ Perc | 787 | 192 | 54 | 1211 | 273 | 19 | > | 0.893 | 0.794 | 0.992 | 1050.1 | 0.944 | 0.789 | 0.904 |
|  | 10^th^ Perc | 856 | 184 | 54 | 1314 | 240 | 19 | > | 0.933 | 0.853 | 1.000 | 1179.9 | 0.981 | 0.842 | 0.945 |
|  | 25^th^ Perc | 972 | 178 | 54 | 1445 | 239 | 19 | > | 0.942 | 0.872 | 1.000 | 1225.0 | 0.944 | 0.895 | 0.932 |
|  | 75^th^ Perc | 1256 | 218 | 54 | 1693 | 288 | 19 | > | 0.896 | 0.814 | 0.977 | 1492.9 | 0.870 | 0.842 | 0.863 |
|  | 90^th^ Perc | 1395 | 236 | 54 | 1799 | 297 | 19 | > | 0.866 | 0.774 | 0.959 | 1573.4 | 0.796 | 0.895 | 0.822 |
|  | 95^th^ Perc | 1493 | 251 | 54 | 1901 | 324 | 19 | > | 0.846 | 0.745 | 0.947 | 1629.9 | 0.704 | 0.895 | 0.753 |
|  | Skewness | 0.4 | 0.7 | 54 | 0.1 | 1.2 | 19 | < | 0.647 | 0.474 | 0.820 | -0.3 | 0.889 | 0.474 | 0.781 |
|  | Kurtosis | 4.2 | 2.1 | 54 | 4.6 | 3.1 | 19 | < | 0.514 | 0.346 | 0.681 | 2.6 | 0.815 | 0.316 | 0.685 |
| **D_1_'** | Mean | 1062 | 175 | 54 | 1463 | 278 | 19 | > | 0.893 | 0.803 | 0.983 | 1292.6 | 0.926 | 0.737 | 0.877 |
|  | Median | 1055 | 182 | 54 | 1471 | 284 | 19 | > | 0.895 | 0.806 | 0.984 | 1311.2 | 0.926 | 0.737 | 0.877 |
|  | Std | 208 | 88 | 54 | 168 | 95 | 19 | < | 0.679 | 0.529 | 0.830 | 202.1 | 0.537 | 0.842 | 0.616 |
|  | 5^th^ Perc | 742 | 190 | 54 | 1173 | 285 | 19 | > | 0.920 | 0.825 | 1.000 | 927.2 | 0.870 | 0.895 | 0.877 |
|  | 10^th^ Perc | 812 | 183 | 54 | 1251 | 246 | 19 | > | 0.951 | 0.904 | 0.999 | 1048.1 | 0.963 | 0.789 | 0.918 |
|  | 25^th^ Perc | 921 | 175 | 54 | 1369 | 253 | 19 | > | 0.944 | 0.888 | 1.000 | 1127.0 | 0.963 | 0.842 | 0.932 |
|  | 75^th^ Perc | 1192 | 211 | 54 | 1560 | 322 | 19 | > | 0.832 | 0.722 | 0.943 | 1409.8 | 0.889 | 0.737 | 0.849 |
|  | 90^th^ Perc | 1326 | 235 | 54 | 1645 | 342 | 19 | > | 0.786 | 0.656 | 0.915 | 1514.8 | 0.852 | 0.737 | 0.822 |
|  | 95^th^ Perc | 1414 | 255 | 54 | 1732 | 358 | 19 | > | 0.766 | 0.630 | 0.902 | 1673.6 | 0.870 | 0.632 | 0.808 |
|  | Skewness | 0.3 | 0.7 | 54 | 0.1 | 1.2 | 19 | < | 0.581 | 0.417 | 0.744 | 0.1 | 0.593 | 0.632 | 0.603 |
|  | Kurtosis | 3.8 | 1.9 | 54 | 4.7 | 3.2 | 19 | > | 0.535 | 0.371 | 0.699 | 5.8 | 0.889 | 0.316 | 0.740 |
| **D_2_'** | Mean | 976 | 189 | 54 | 1310 | 318 | 19 | > | 0.816 | 0.699 | 0.932 | 1183.8 | 0.870 | 0.632 | 0.808 |
|  | Median | 967 | 193 | 54 | 1312 | 330 | 19 | > | 0.812 | 0.694 | 0.930 | 1209.1 | 0.907 | 0.579 | 0.822 |
|  | Std | 215 | 87 | 54 | 170 | 68 | 19 | < | 0.658 | 0.515 | 0.801 | 219.9 | 0.481 | 0.895 | 0.589 |
|  | 5^th^ Perc | 644 | 204 | 54 | 1024 | 318 | 19 | > | 0.856 | 0.762 | 0.949 | 836.7 | 0.833 | 0.737 | 0.808 |
|  | 10^th^ Perc | 715 | 197 | 54 | 1102 | 311 | 19 | > | 0.850 | 0.741 | 0.959 | 989.9 | 0.963 | 0.632 | 0.877 |
|  | 25^th^ Perc | 831 | 186 | 54 | 1209 | 320 | 19 | > | 0.849 | 0.739 | 0.959 | 1071.3 | 0.944 | 0.684 | 0.877 |
|  | 75^th^ Perc | 1113 | 222 | 54 | 1418 | 333 | 19 | > | 0.762 | 0.639 | 0.886 | 1105.2 | 0.537 | 0.842 | 0.616 |
|  | 90^th^ Perc | 1253 | 252 | 54 | 1515 | 333 | 19 | > | 0.731 | 0.605 | 0.857 | 1326.7 | 0.648 | 0.737 | 0.671 |
|  | 95^th^ Perc | 1341 | 266 | 54 | 1581 | 333 | 19 | > | 0.710 | 0.576 | 0.844 | 1364.9 | 0.574 | 0.737 | 0.616 |
|  | Skewness | 0.3 | 0.7 | 54 | 0.1 | 1.0 | 19 | < | 0.563 | 0.402 | 0.724 | 0.0 | 0.667 | 0.526 | 0.630 |
|  | Kurtosis | 3.8 | 2.4 | 54 | 4.1 | 2.6 | 19 | < | 0.506 | 0.348 | 0.664 | 3.5 | 0.426 | 0.737 | 0.507 |
| **f_1_'** | Mean | 59 | 39 | 54 | 98 | 66 | 19 | > | 0.662 | 0.494 | 0.830 | 96.6 | 0.870 | 0.526 | 0.781 |
|  | Median | 44 | 42 | 54 | 86 | 69 | 19 | > | 0.663 | 0.496 | 0.829 | 100.0 | 0.926 | 0.474 | 0.808 |
|  | Std | 58 | 26 | 54 | 57 | 30 | 19 | > | 0.510 | 0.351 | 0.668 | 58.2 | 0.648 | 0.474 | 0.603 |
|  | 5^th^ Perc | 2 | 8 | 54 | 30 | 42 | 19 | > | 0.681 | 0.555 | 0.806 | 18.9 | 0.981 | 0.421 | 0.836 |
|  | 10^th^ Perc | 4 | 13 | 54 | 40 | 50 | 19 | > | 0.734 | 0.603 | 0.865 | 12.6 | 0.907 | 0.526 | 0.808 |
|  | 25^th^ Perc | 15 | 26 | 54 | 58 | 60 | 19 | > | 0.684 | 0.528 | 0.840 | 70.4 | 0.981 | 0.474 | 0.849 |
|  | 75^th^ Perc | 89 | 60 | 54 | 127 | 81 | 19 | > | 0.645 | 0.477 | 0.813 | 172.5 | 0.944 | 0.421 | 0.808 |
|  | 90^th^ Perc | 135 | 74 | 54 | 174 | 99 | 19 | > | 0.624 | 0.452 | 0.796 | 214.6 | 0.889 | 0.474 | 0.781 |
|  | 95^th^ Perc | 169 | 82 | 54 | 206 | 111 | 19 | > | 0.610 | 0.438 | 0.782 | 226.7 | 0.852 | 0.474 | 0.753 |
|  | Skewness | 1.6 | 1.2 | 54 | 1.0 | 1.3 | 18 | < | 0.654 | 0.495 | 0.814 | 0.8 | 0.741 | 0.611 | 0.708 |
|  | Kurtosis | 7.5 | 7.9 | 54 | 6.2 | 6.3 | 18 | < | 0.569 | 0.415 | 0.723 | 5.1 | 0.500 | 0.778 | 0.569 |
| **f_2_'** | Mean | 118 | 76 | 54 | 188 | 118 | 19 | > | 0.667 | 0.501 | 0.832 | 172.1 | 0.833 | 0.579 | 0.767 |
|  | Median | 105 | 88 | 54 | 184 | 127 | 19 | > | 0.678 | 0.522 | 0.835 | 169.8 | 0.833 | 0.579 | 0.767 |
|  | Std | 81 | 28 | 54 | 82 | 39 | 19 | < | 0.511 | 0.346 | 0.676 | 81.0 | 0.500 | 0.632 | 0.534 |
|  | 5^th^ Perc | 19 | 40 | 54 | 68 | 73 | 19 | > | 0.702 | 0.562 | 0.842 | 54.3 | 0.870 | 0.526 | 0.781 |
|  | 10^th^ Perc | 30 | 52 | 54 | 89 | 87 | 19 | > | 0.699 | 0.554 | 0.843 | 97.8 | 0.889 | 0.526 | 0.795 |
|  | 25^th^ Perc | 58 | 71 | 54 | 129 | 109 | 19 | > | 0.683 | 0.526 | 0.840 | 125.7 | 0.833 | 0.579 | 0.767 |
|  | 75^th^ Perc | 164 | 95 | 54 | 236 | 139 | 19 | > | 0.664 | 0.502 | 0.827 | 214.8 | 0.815 | 0.579 | 0.753 |
|  | 90^th^ Perc | 225 | 99 | 54 | 293 | 154 | 19 | > | 0.646 | 0.480 | 0.812 | 325.4 | 0.889 | 0.421 | 0.767 |
|  | 95^th^ Perc | 263 | 100 | 54 | 328 | 162 | 19 | > | 0.635 | 0.468 | 0.801 | 364.3 | 0.889 | 0.421 | 0.767 |
|  | Skewness | 1.0 | 1.2 | 54 | 0.9 | 2.1 | 19 | < | 0.629 | 0.475 | 0.782 | 0.2 | 0.796 | 0.474 | 0.712 |
|  | Kurtosis | 5.6 | 5.6 | 54 | 8.3 | 19.0 | 19 | < | 0.566 | 0.417 | 0.715 | 4.1 | 0.519 | 0.737 | 0.575 |
| **D*'** | Mean | 17273 | 7256 | 53 | 19740 | 10820 | 17 | > | 0.563 | 0.389 | 0.736 | 21309.9 | 0.774 | 0.412 | 0.686 |
|  | Median | 13960 | 6525 | 53 | 17370 | 10122 | 17 | > | 0.605 | 0.433 | 0.777 | 13334.3 | 0.491 | 0.765 | 0.557 |
|  | Std | 13510 | 5181 | 53 | 11671 | 6822 | 17 | < | 0.573 | 0.399 | 0.746 | 7489.8 | 0.906 | 0.353 | 0.771 |
|  | 5^th^ Perc | 3237 | 2254 | 53 | 7805 | 7484 | 17 | > | 0.727 | 0.563 | 0.891 | 4183.6 | 0.849 | 0.647 | 0.800 |
|  | 10^th^ Perc | 4632 | 2687 | 53 | 9416 | 7619 | 17 | > | 0.721 | 0.552 | 0.891 | 6033.0 | 0.849 | 0.647 | 0.800 |
|  | 25^th^ Perc | 8348 | 4174 | 53 | 12441 | 7736 | 17 | > | 0.674 | 0.498 | 0.850 | 10221.4 | 0.792 | 0.647 | 0.757 |
|  | 75^th^ Perc | 22124 | 10145 | 53 | 24006 | 13117 | 17 | > | 0.541 | 0.369 | 0.712 | 42936.4 | 0.981 | 0.176 | 0.786 |
|  | 90^th^ Perc | 33581 | 14796 | 53 | 32675 | 17715 | 17 | < | 0.507 | 0.341 | 0.673 | 10943.4 | 0.962 | 0.176 | 0.771 |
|  | 95^th^ Perc | 43443 | 18117 | 53 | 39887 | 23446 | 17 | < | 0.560 | 0.395 | 0.726 | 51579.5 | 0.302 | 0.882 | 0.443 |
|  | Skewness | 2.2 | 1.2 | 53 | 2.1 | 1.3 | 17 | < | 0.536 | 0.373 | 0.700 | 2.2 | 0.434 | 0.765 | 0.514 |
|  | Kurtosis | 12.0 | 14.0 | 53 | 11.6 | 11.6 | 17 | < | 0.529 | 0.363 | 0.696 | 10.0 | 0.453 | 0.706 | 0.514 |
|  |  |  |  |  |  |  |  |  |  |  |  |  |  |  |  |
|  |  |  |  |  |  |  |  |  |  |  |  |  |  |  |  |
| **ROIs excluding necrosis, cystic components, scars** | | | | | | | | | | | | | | | |
| **ADC** | Mean | 1090 | 167 | 54 | 1566 | 251 | 19 | > | 0.953 | 0.891 | 1.000 | 1276.1 | 0.870 | 0.947 | 0.890 |
|  | Median | 1080 | 178 | 54 | 1573 | 255 | 19 | > | 0.955 | 0.895 | 1.000 | 1321.9 | 0.926 | 0.895 | 0.918 |
|  | Std | 203 | 75 | 54 | 206 | 82 | 19 | < | 0.509 | 0.353 | 0.665 | 212.7 | 0.481 | 0.684 | 0.534 |
|  | 5^th^ Perc | 779 | 197 | 54 | 1211 | 273 | 19 | > | 0.895 | 0.797 | 0.993 | 1048.1 | 0.944 | 0.789 | 0.904 |
|  | 10^th^ Perc | 846 | 187 | 54 | 1314 | 240 | 19 | > | 0.939 | 0.864 | 1.000 | 1179.9 | 0.981 | 0.842 | 0.945 |
|  | 25^th^ Perc | 957 | 177 | 54 | 1445 | 239 | 19 | > | 0.946 | 0.878 | 1.000 | 1225.0 | 0.963 | 0.895 | 0.945 |
|  | 75^th^ Perc | 1212 | 181 | 54 | 1693 | 288 | 19 | > | 0.939 | 0.869 | 1.000 | 1492.9 | 0.963 | 0.842 | 0.932 |
|  | 90^th^ Perc | 1341 | 191 | 54 | 1799 | 297 | 19 | > | 0.917 | 0.842 | 0.992 | 1573.4 | 0.870 | 0.895 | 0.877 |
|  | 95^th^ Perc | 1433 | 204 | 54 | 1901 | 324 | 19 | > | 0.903 | 0.817 | 0.988 | 1630.1 | 0.815 | 0.895 | 0.836 |
|  | Skewness | 0.4 | 0.7 | 54 | 0.1 | 1.2 | 19 | < | 0.644 | 0.469 | 0.819 | 0.0 | 0.741 | 0.632 | 0.712 |
|  | Kurtosis | 4.3 | 2.0 | 54 | 4.6 | 3.1 | 19 | < | 0.541 | 0.369 | 0.712 | 2.8 | 0.833 | 0.368 | 0.712 |
| **D_1_'** | Mean | 1032 | 156 | 54 | 1463 | 278 | 19 | > | 0.920 | 0.843 | 0.997 | 1214.9 | 0.944 | 0.789 | 0.904 |
|  | Median | 1025 | 164 | 54 | 1471 | 284 | 19 | > | 0.918 | 0.841 | 0.995 | 1311.2 | 0.981 | 0.737 | 0.918 |
|  | Std | 189 | 80 | 54 | 168 | 95 | 19 | < | 0.636 | 0.479 | 0.794 | 144.5 | 0.704 | 0.579 | 0.671 |
|  | 5^th^ Perc | 736 | 191 | 54 | 1173 | 285 | 19 | > | 0.923 | 0.829 | 1.000 | 892.9 | 0.833 | 0.947 | 0.863 |
|  | 10^th^ Perc | 803 | 183 | 54 | 1251 | 246 | 19 | > | 0.957 | 0.913 | 1.000 | 1048.1 | 0.981 | 0.789 | 0.932 |
|  | 25^th^ Perc | 908 | 175 | 54 | 1369 | 253 | 19 | > | 0.950 | 0.897 | 1.000 | 1124.5 | 0.981 | 0.842 | 0.945 |
|  | 75^th^ Perc | 1146 | 169 | 54 | 1560 | 322 | 19 | > | 0.877 | 0.778 | 0.977 | 1405.7 | 0.963 | 0.737 | 0.904 |
|  | 90^th^ Perc | 1272 | 188 | 54 | 1645 | 342 | 19 | > | 0.838 | 0.719 | 0.957 | 1514.8 | 0.926 | 0.737 | 0.877 |
|  | 95^th^ Perc | 1352 | 206 | 54 | 1732 | 358 | 19 | > | 0.822 | 0.695 | 0.949 | 1578.6 | 0.870 | 0.737 | 0.836 |
|  | Skewness | 0.3 | 0.7 | 54 | 0.1 | 1.2 | 19 | < | 0.574 | 0.409 | 0.739 | 0.1 | 0.593 | 0.632 | 0.603 |
|  | Kurtosis | 3.9 | 1.8 | 54 | 4.7 | 3.2 | 19 | > | 0.505 | 0.333 | 0.676 | 5.8 | 0.907 | 0.316 | 0.753 |
| **D_2_'** | Mean | 945 | 171 | 54 | 1310 | 318 | 19 | > | 0.852 | 0.747 | 0.957 | 1183.8 | 0.963 | 0.632 | 0.877 |
|  | Median | 936 | 178 | 54 | 1312 | 330 | 19 | > | 0.843 | 0.734 | 0.952 | 1227.5 | 0.981 | 0.579 | 0.877 |
|  | Std | 197 | 75 | 54 | 170 | 68 | 19 | < | 0.614 | 0.465 | 0.763 | 184.8 | 0.556 | 0.737 | 0.603 |
|  | 5^th^ Perc | 635 | 203 | 54 | 1024 | 318 | 19 | > | 0.865 | 0.778 | 0.953 | 825.3 | 0.833 | 0.737 | 0.808 |
|  | 10^th^ Perc | 705 | 197 | 54 | 1102 | 311 | 19 | > | 0.860 | 0.753 | 0.966 | 851.3 | 0.815 | 0.789 | 0.808 |
|  | 25^th^ Perc | 817 | 185 | 54 | 1209 | 320 | 19 | > | 0.858 | 0.751 | 0.964 | 1068.1 | 0.944 | 0.684 | 0.877 |
|  | 75^th^ Perc | 1067 | 183 | 54 | 1418 | 333 | 19 | > | 0.817 | 0.703 | 0.930 | 1232.5 | 0.796 | 0.684 | 0.767 |
|  | 90^th^ Perc | 1198 | 204 | 54 | 1515 | 333 | 19 | > | 0.789 | 0.673 | 0.906 | 1326.7 | 0.759 | 0.737 | 0.753 |
|  | 95^th^ Perc | 1279 | 212 | 54 | 1581 | 333 | 19 | > | 0.766 | 0.639 | 0.893 | 1547.6 | 0.889 | 0.526 | 0.795 |
|  | Skewness | 0.2 | 0.7 | 54 | 0.1 | 1.0 | 19 | < | 0.535 | 0.371 | 0.699 | -0.1 | 0.704 | 0.474 | 0.644 |
|  | Kurtosis | 3.9 | 2.3 | 54 | 4.1 | 2.6 | 19 | < | 0.541 | 0.378 | 0.704 | 3.5 | 0.463 | 0.737 | 0.534 |
| **f_1_'** | Mean | 59 | 38 | 54 | 98 | 66 | 19 | > | 0.661 | 0.493 | 0.829 | 96.6 | 0.870 | 0.526 | 0.781 |
|  | Median | 44 | 42 | 54 | 86 | 69 | 19 | > | 0.665 | 0.499 | 0.830 | 116.3 | 0.963 | 0.421 | 0.822 |
|  | Std | 58 | 26 | 54 | 57 | 30 | 19 | < | 0.500 | 0.342 | 0.658 |  |  |  |  |
|  | 5^th^ Perc | 2 | 8 | 54 | 30 | 42 | 19 | > | 0.681 | 0.555 | 0.806 | 18.9 | 0.981 | 0.421 | 0.836 |
|  | 10^th^ Perc | 4 | 13 | 54 | 40 | 50 | 19 | > | 0.734 | 0.603 | 0.865 | 12.6 | 0.907 | 0.526 | 0.808 |
|  | 25^th^ Perc | 15 | 26 | 54 | 58 | 60 | 19 | > | 0.685 | 0.529 | 0.841 | 70.4 | 0.981 | 0.474 | 0.849 |
|  | 75^th^ Perc | 90 | 60 | 54 | 127 | 81 | 19 | > | 0.644 | 0.475 | 0.813 | 173.7 | 0.944 | 0.421 | 0.808 |
|  | 90^th^ Perc | 136 | 73 | 54 | 174 | 99 | 19 | > | 0.626 | 0.454 | 0.799 | 214.6 | 0.889 | 0.474 | 0.781 |
|  | 95^th^ Perc | 169 | 80 | 54 | 206 | 111 | 19 | > | 0.605 | 0.431 | 0.779 | 226.7 | 0.852 | 0.474 | 0.753 |
|  | Skewness | 1.6 | 1.2 | 54 | 1.0 | 1.3 | 18 | < | 0.652 | 0.492 | 0.813 | 0.8 | 0.722 | 0.611 | 0.694 |
|  | Kurtosis | 7.3 | 7.7 | 54 | 6.2 | 6.3 | 18 | < | 0.568 | 0.414 | 0.722 | 5.1 | 0.481 | 0.778 | 0.556 |
| **f_2_'** | Mean | 119 | 76 | 54 | 188 | 118 | 19 | > | 0.666 | 0.499 | 0.832 | 172.1 | 0.833 | 0.579 | 0.767 |
|  | Median | 105 | 88 | 54 | 184 | 127 | 19 | > | 0.680 | 0.524 | 0.836 | 169.8 | 0.833 | 0.579 | 0.767 |
|  | Std | 82 | 29 | 54 | 82 | 39 | 19 | < | 0.520 | 0.357 | 0.684 | 81.0 | 0.519 | 0.632 | 0.548 |
|  | 5^th^ Perc | 19 | 40 | 54 | 68 | 73 | 19 | > | 0.701 | 0.562 | 0.841 | 10.2 | 0.759 | 0.632 | 0.726 |
|  | 10^th^ Perc | 30 | 52 | 54 | 89 | 87 | 19 | > | 0.697 | 0.553 | 0.841 | 97.8 | 0.870 | 0.526 | 0.781 |
|  | 25^th^ Perc | 59 | 72 | 54 | 129 | 109 | 19 | > | 0.681 | 0.525 | 0.838 | 125.7 | 0.833 | 0.579 | 0.767 |
|  | 75^th^ Perc | 166 | 95 | 54 | 236 | 139 | 19 | > | 0.660 | 0.498 | 0.823 | 214.8 | 0.796 | 0.579 | 0.740 |
|  | 90^th^ Perc | 227 | 99 | 54 | 293 | 154 | 19 | > | 0.640 | 0.474 | 0.806 | 325.4 | 0.889 | 0.421 | 0.767 |
|  | 95^th^ Perc | 265 | 102 | 54 | 328 | 162 | 19 | > | 0.629 | 0.462 | 0.795 | 359.6 | 0.870 | 0.421 | 0.753 |
|  | Skewness | 1.1 | 1.3 | 54 | 0.9 | 2.1 | 19 | < | 0.633 | 0.479 | 0.786 | 0.2 | 0.796 | 0.474 | 0.712 |
|  | Kurtosis | 6.0 | 7.9 | 54 | 8.3 | 19.0 | 19 | < | 0.567 | 0.419 | 0.715 | 4.1 | 0.519 | 0.737 | 0.575 |
| **D*'** | Mean | 17895 | 8443 | 54 | 19740 | 10820 | 17 | > | 0.559 | 0.388 | 0.730 | 21309.9 | 0.759 | 0.412 | 0.676 |
|  | Median | 14605 | 8170 | 54 | 17370 | 10122 | 17 | > | 0.597 | 0.427 | 0.767 | 13334.3 | 0.500 | 0.765 | 0.563 |
|  | Std | 13373 | 5180 | 54 | 11671 | 6822 | 17 | < | 0.572 | 0.399 | 0.745 | 7434.7 | 0.889 | 0.353 | 0.761 |
|  | 5^th^ Perc | 4069 | 6454 | 54 | 7805 | 7484 | 17 | > | 0.717 | 0.555 | 0.879 | 4183.6 | 0.833 | 0.647 | 0.789 |
|  | 10^th^ Perc | 5439 | 6496 | 54 | 9416 | 7619 | 17 | > | 0.710 | 0.542 | 0.878 | 6033.0 | 0.833 | 0.647 | 0.789 |
|  | 25^th^ Perc | 9052 | 6739 | 54 | 12441 | 7736 | 17 | > | 0.661 | 0.486 | 0.837 | 10221.4 | 0.778 | 0.647 | 0.746 |
|  | 75^th^ Perc | 22677 | 10743 | 54 | 24006 | 13117 | 17 | > | 0.536 | 0.366 | 0.706 | 22859.7 | 0.556 | 0.588 | 0.563 |
|  | 90^th^ Perc | 34023 | 14743 | 54 | 32675 | 17715 | 17 | < | 0.511 | 0.344 | 0.678 | 10943.4 | 0.963 | 0.176 | 0.775 |
|  | 95^th^ Perc | 43723 | 17790 | 54 | 39887 | 23446 | 17 | < | 0.562 | 0.396 | 0.728 | 51579.5 | 0.315 | 0.882 | 0.451 |
|  | Skewness | 2.1 | 1.1 | 54 | 2.1 | 1.3 | 17 | < | 0.501 | 0.338 | 0.664 | 2.2 | 0.389 | 0.765 | 0.479 |
|  | Kurtosis | 11.4 | 13.7 | 54 | 11.6 | 11.6 | 17 | < | 0.509 | 0.344 | 0.674 | 10.0 | 0.426 | 0.706 | 0.493 |

V – value of histogram metric, SD – standard deviation, N – number of cases, Dir - test direction (">" / "<" means that a lower / higher test result indicates a more positive test), AUC - area under the curve, CI - confidence interval, Sen - sensitivity (true positive rate), Spec - specificity (true negative rate), Acc - accuracy (rate of correctly identified cases), Std - standard deviation, Perc - percentile

**Supplementary table 2.** Comparison of AUC values of the ROC curves obtained from mean values (1) and from other histogram metrics (2) of the parameters (see Table 4) at 1.5 T (a) and 3.0 T (b). Improved AUC values are given in bold type. Significant results are marked by star.

a) 1.5 T

| **Par** | **1** | **2** | **AUC1** | **AUC2** | **P** |  | **AUC1** | **AUC2** | **P** |  | **AUC1** | **AUC2** | **P** |  |
| --- | --- | --- | --- | --- | --- | --- | --- | --- | --- | --- | --- | --- | --- | --- |
|  |  |  | **Incl** | **Incl** |  |  | **Excl** | **Incl** |  |  | **Excl** | **Excl** |  |  |
| **ADC** | MV | Median | 0.925 | **0.928** | 5.5E-01 |  | 0.958 | 0.928 | 1.1E-02 | * | 0.958 | 0.955 | 3.5E-01 |  |
|  | MV | Std | 0.925 | 0.597 | 7.2E-06 | * | 0.958 | 0.597 | 1.3E-07 | * | 0.958 | 0.543 | 8.2E-09 | * |
|  | MV | P5 | 0.925 | **0.971** | 2.3E-02 | * | 0.958 | **0.971** | 3.3E-01 |  | 0.958 | **0.974** | 2.6E-01 |  |
|  | MV | P10 | 0.925 | **0.969** | 1.8E-02 | * | 0.958 | **0.969** | 3.4E-01 |  | 0.958 | **0.975** | 1.3E-01 |  |
|  | MV | P25 | 0.925 | **0.956** | 1.2E-02 | * | 0.958 | 0.956 | 7.5E-01 |  | 0.958 | **0.968** | 1.3E-01 |  |
|  | MV | P75 | 0.925 | 0.874 | 2.6E-05 | * | 0.958 | 0.874 | 2.2E-04 | * | 0.958 | 0.933 | 3.4E-03 | * |
|  | MV | P90 | 0.925 | 0.831 | 3.0E-06 | * | 0.958 | 0.831 | 2.3E-05 | * | 0.958 | 0.904 | 9.3E-04 | * |
|  | MV | P95 | 0.925 | 0.815 | 3.8E-06 | * | 0.958 | 0.815 | 1.6E-05 | * | 0.958 | 0.894 | 1.0E-03 | * |
|  | MV | Skewness | 0.925 | 0.566 | 2.0E-10 | * | 0.958 | 0.566 | 9.6E-12 | * | 0.958 | 0.553 | 5.0E-12 | * |
|  | MV | Kurtosis | 0.925 | 0.594 | 1.5E-06 | * | 0.958 | 0.594 | 2.8E-08 | * | 0.958 | 0.610 | 1.0E-07 | * |
| **D_1_'** | MV | Median | 0.866 | **0.872** | 3.6E-01 |  | 0.902 | 0.872 | 1.1E-02 | * | 0.902 | **0.905** | 5.2E-01 |  |
|  | MV | Std | 0.866 | 0.614 | 2.5E-03 | * | 0.902 | 0.614 | 1.4E-04 | * | 0.902 | 0.553 | 1.5E-05 | * |
|  | MV | P5 | 0.866 | **0.927** | 9.0E-03 | * | 0.902 | **0.927** | 1.8E-01 |  | 0.902 | **0.934** | 9.6E-02 |  |
|  | MV | P10 | 0.866 | **0.926** | 4.2E-03 | * | 0.902 | **0.926** | 1.2E-01 |  | 0.902 | **0.935** | 3.8E-02 | * |
|  | MV | P25 | 0.866 | **0.910** | 1.6E-03 | * | 0.902 | **0.910** | 3.8E-01 |  | 0.902 | **0.929** | 1.1E-02 | * |
|  | MV | P75 | 0.866 | 0.807 | 1.5E-05 | * | 0.902 | 0.807 | 3.4E-05 | * | 0.902 | 0.865 | 4.4E-03 | * |
|  | MV | P90 | 0.866 | 0.754 | 8.1E-07 | * | 0.902 | 0.754 | 3.7E-06 | * | 0.902 | 0.832 | 6.0E-04 | * |
|  | MV | P95 | 0.866 | 0.736 | 2.0E-06 | * | 0.902 | 0.736 | 3.9E-06 | * | 0.902 | 0.811 | 4.2E-04 | * |
|  | MV | Skewness | 0.866 | 0.667 | 1.8E-03 | * | 0.902 | 0.667 | 1.4E-04 | * | 0.902 | 0.648 | 8.7E-05 | * |
|  | MV | Kurtosis | 0.866 | 0.629 | 1.0E-03 | * | 0.902 | 0.629 | 6.7E-05 | * | 0.902 | 0.627 | 8.0E-05 | * |
| **D_2_'** | MV | Median | 0.822 | **0.832** | 1.8E-01 |  | 0.864 | 0.832 | 8.7E-03 | * | 0.864 | 0.862 | 5.9E-01 |  |
|  | MV | Std | 0.822 | 0.591 | 2.8E-03 | * | 0.864 | 0.591 | 2.0E-04 | * | 0.864 | 0.527 | 1.4E-05 | * |
|  | MV | P5 | 0.822 | **0.841** | 5.7E-01 |  | 0.864 | 0.841 | 4.5E-01 |  | 0.864 | 0.844 | 5.1E-01 |  |
|  | MV | P10 | 0.822 | **0.856** | 7.4E-02 |  | 0.864 | 0.856 | 6.4E-01 |  | 0.864 | 0.861 | 8.7E-01 |  |
|  | MV | P25 | 0.822 | **0.852** | 1.9E-02 | * | 0.864 | 0.852 | 2.9E-01 |  | 0.864 | 0.863 | 8.9E-01 |  |
|  | MV | P75 | 0.822 | 0.792 | 2.4E-02 | * | 0.864 | 0.792 | 4.6E-04 | * | 0.864 | 0.851 | 1.3E-01 |  |
|  | MV | P90 | 0.822 | 0.753 | 1.1E-03 | * | 0.864 | 0.753 | 6.6E-05 | * | 0.864 | 0.830 | 2.3E-02 | * |
|  | MV | P95 | 0.822 | 0.720 | 1.5E-04 | * | 0.864 | 0.720 | 1.5E-05 | * | 0.864 | 0.807 | 6.6E-03 | * |
|  | MV | Skewness | 0.822 | 0.620 | 5.4E-03 | * | 0.864 | 0.620 | 3.8E-04 | * | 0.864 | 0.595 | 8.1E-05 | * |
|  | MV | Kurtosis | 0.822 | 0.571 | 7.9E-04 | * | 0.864 | 0.571 | 3.6E-05 | * | 0.864 | 0.557 | 1.6E-05 | * |
| **f_1_'** | MV | Median | 0.621 | 0.620 | 9.7E-01 |  | 0.622 | 0.620 | 9.3E-01 |  | 0.622 | 0.615 | 7.3E-01 |  |
|  | MV | Std | 0.621 | 0.507 | 3.1E-01 |  | 0.622 | 0.507 | 3.2E-01 |  | 0.622 | 0.512 | 6.1E-02 |  |
|  | MV | P5 | 0.621 | **0.712** | 2.8E-02 | * | 0.622 | **0.712** | 3.1E-02 | * | 0.622 | **0.708** | 3.4E-02 | * |
|  | MV | P10 | 0.621 | **0.671** | 2.0E-01 |  | 0.622 | **0.671** | 2.1E-01 |  | 0.622 | **0.675** | 1.7E-01 |  |
|  | MV | P25 | 0.621 | **0.637** | 6.6E-01 |  | 0.622 | **0.637** | 6.9E-01 |  | 0.622 | **0.638** | 6.7E-01 |  |
|  | MV | P75 | 0.621 | 0.606 | 1.6E-01 |  | 0.622 | 0.606 | 1.8E-01 |  | 0.622 | 0.603 | 8.7E-02 |  |
|  | MV | P90 | 0.621 | 0.612 | 7.5E-01 |  | 0.622 | 0.612 | 7.2E-01 |  | 0.622 | 0.612 | 7.1E-01 |  |
|  | MV | P95 | 0.621 | 0.594 | 4.3E-01 |  | 0.622 | 0.594 | 4.2E-01 |  | 0.622 | 0.604 | 5.8E-01 |  |
|  | MV | Skewness | 0.621 | 0.539 | 1.2E-01 |  | 0.622 | 0.539 | 1.2E-01 |  | 0.622 | 0.507 | 3.5E-02 | * |
|  | MV | Kurtosis | 0.621 | 0.531 | 1.6E-01 |  | 0.622 | 0.531 | 1.6E-01 |  | 0.622 | 0.514 | 3.2E-01 |  |
| **f_2_'** | MV | Median | 0.656 | 0.637 | 9.8E-02 |  | 0.672 | 0.637 | 3.3E-02 | * | 0.672 | 0.660 | 2.6E-01 |  |
|  | MV | Std | 0.656 | 0.534 | 1.1E-01 |  | 0.672 | 0.534 | 7.2E-02 |  | 0.672 | 0.553 | 1.1E-01 |  |
|  | MV | P5 | 0.656 | 0.637 | 6.2E-01 |  | 0.672 | 0.637 | 3.8E-01 |  | 0.672 | 0.655 | 6.7E-01 |  |
|  | MV | P10 | 0.656 | 0.639 | 6.5E-01 |  | 0.672 | 0.639 | 4.0E-01 |  | 0.672 | 0.653 | 6.2E-01 |  |
|  | MV | P25 | 0.656 | 0.633 | 3.6E-01 |  | 0.672 | 0.633 | 1.6E-01 |  | 0.672 | 0.649 | 3.4E-01 |  |
|  | MV | P75 | 0.656 | 0.651 | 7.2E-01 |  | 0.672 | 0.651 | 2.9E-01 |  | 0.672 | 0.668 | 8.2E-01 |  |
|  | MV | P90 | 0.656 | 0.654 | 9.6E-01 |  | 0.672 | 0.654 | 6.2E-01 |  | 0.672 | 0.661 | 7.6E-01 |  |
|  | MV | P95 | 0.656 | 0.642 | 7.4E-01 |  | 0.672 | 0.642 | 4.9E-01 |  | 0.672 | 0.643 | 4.9E-01 |  |
|  | MV | Skewness | 0.656 | 0.564 | 3.8E-02 | * | 0.672 | 0.564 | 1.7E-02 | * | 0.672 | 0.558 | 1.1E-02 | * |
|  | MV | Kurtosis | 0.656 | 0.553 | 1.0E-01 |  | 0.672 | 0.553 | 6.3E-02 |  | 0.672 | 0.510 | 1.1E-02 | * |
| **D*'** | MV | Median | 0.529 | **0.545** | 3.0E-01 |  | 0.515 | **0.545** | 9.1E-02 |  | 0.515 | **0.534** | 1.9E-01 |  |
|  | MV | Std | 0.529 | **0.547** | 8.8E-01 |  | 0.515 | **0.547** | 8.0E-01 |  | 0.515 | **0.551** | 7.7E-01 |  |
|  | MV | P5 | 0.529 | **0.658** | 1.2E-03 | * | 0.515 | **0.658** | 3.4E-04 | * | 0.515 | **0.646** | 8.5E-04 | * |
|  | MV | P10 | 0.529 | **0.617** | 1.2E-02 | * | 0.515 | **0.617** | 4.5E-03 | * | 0.515 | **0.608** | 9.5E-03 | * |
|  | MV | P25 | 0.529 | **0.558** | 2.8E-01 |  | 0.515 | **0.558** | 1.2E-01 |  | 0.515 | **0.549** | 2.2E-01 |  |
|  | MV | P75 | 0.529 | **0.532** | 7.3E-01 |  | 0.515 | **0.532** | 2.4E-01 |  | 0.515 | **0.517** | 8.6E-01 |  |
|  | MV | P90 | 0.529 | 0.515 | 4.1E-01 |  | 0.515 | **0.515** | 9.7E-01 |  | 0.515 | 0.508 | 7.2E-01 |  |
|  | MV | P95 | 0.529 | 0.519 | 9.4E-01 |  | 0.515 | **0.519** | 9.7E-01 |  | 0.515 | **0.525** | 9.3E-01 |  |
|  | MV | Skewness | 0.529 | 0.679 | 9.0E-02 |  | 0.515 | **0.679** | 5.7E-02 |  | 0.515 | **0.652** | 1.2E-01 |  |
|  | MV | Kurtosis | 0.529 | 0.686 | 6.8E-02 |  | 0.515 | **0.686** | 5.1E-02 |  | 0.515 | **0.654** | 1.2E-01 |  |

b) 3.0 T

| **Par** | **1** | **2** | **AUC1** | **AUC2** | **P** |  | **AUC1** | **AUC2** | **P** |  | **AUC1** | **AUC2** | **P** |  |
| --- | --- | --- | --- | --- | --- | --- | --- | --- | --- | --- | --- | --- | --- | --- |
|  |  |  | **Incl** | **Incl** |  |  | **Excl** | **Incl** |  |  | **Excl** | **Excl** |  |  |
| **ADC** | MV | Median | 0.931 | **0.937** | 3.9E-01 |  | 0.953 | 0.937 | 1.4E-01 |  | 0.953 | **0.955** | 6.4E-01 |  |
|  | MV | Std | 0.931 | 0.578 | 1.5E-04 | * | 0.953 | 0.578 | 8.8E-06 | * | 0.953 | 0.509 | 2.5E-07 | * |
|  | MV | P5 | 0.931 | 0.893 | 2.9E-01 |  | 0.953 | 0.893 | 8.3E-02 |  | 0.953 | 0.895 | 8.9E-02 |  |
|  | MV | P10 | 0.931 | **0.933** | 9.2E-01 |  | 0.953 | 0.933 | 2.7E-01 |  | 0.953 | 0.939 | 3.5E-01 |  |
|  | MV | P25 | 0.931 | **0.942** | 5.0E-01 |  | 0.953 | 0.942 | 5.1E-01 |  | 0.953 | 0.946 | 6.4E-01 |  |
|  | MV | P75 | 0.931 | 0.896 | 6.9E-02 |  | 0.953 | 0.896 | 3.2E-02 | * | 0.953 | 0.939 | 3.7E-01 |  |
|  | MV | P90 | 0.931 | 0.866 | 1.5E-02 | * | 0.953 | 0.866 | 1.1E-02 | * | 0.953 | 0.917 | 1.2E-01 |  |
|  | MV | P95 | 0.931 | 0.846 | 1.8E-03 | * | 0.953 | 0.846 | 2.4E-03 | * | 0.953 | 0.903 | 3.1E-02 | * |
|  | MV | Skewness | 0.931 | 0.647 | 5.6E-03 | * | 0.953 | 0.647 | 2.3E-03 | * | 0.953 | 0.644 | 1.7E-03 | * |
|  | MV | Kurtosis | 0.931 | 0.514 | 2.4E-06 | * | 0.953 | 0.514 | 4.9E-07 | * | 0.953 | 0.541 | 3.7E-06 | * |
| **D_1_'** | MV | Median | 0.893 | **0.895** | 7.8E-01 |  | 0.920 | 0.895 | 4.7E-02 | * | 0.920 | 0.918 | 7.0E-01 |  |
|  | MV | Std | 0.893 | 0.679 | 3.5E-02 | * | 0.920 | 0.679 | 1.3E-02 | * | 0.920 | 0.636 | 3.9E-03 | * |
|  | MV | P5 | 0.893 | **0.920** | 6.4E-01 |  | 0.920 | 0.920 | 1.0E+00 |  | 0.920 | **0.923** | 9.6E-01 |  |
|  | MV | P10 | 0.893 | **0.951** | 8.8E-02 |  | 0.920 | **0.951** | 3.0E-01 |  | 0.920 | **0.957** | 2.4E-01 |  |
|  | MV | P25 | 0.893 | **0.944** | 1.4E-02 | * | 0.920 | **0.944** | 9.8E-02 |  | 0.920 | **0.950** | 4.8E-02 | * |
|  | MV | P75 | 0.893 | 0.832 | 8.4E-04 | * | 0.920 | 0.832 | 1.6E-03 | * | 0.920 | 0.877 | 1.6E-02 | * |
|  | MV | P90 | 0.893 | 0.786 | 2.4E-04 | * | 0.920 | 0.786 | 5.2E-04 | * | 0.920 | 0.838 | 6.6E-03 | * |
|  | MV | P95 | 0.893 | 0.766 | 1.7E-04 | * | 0.920 | 0.766 | 2.9E-04 | * | 0.920 | 0.822 | 4.5E-03 | * |
|  | MV | Skewness | 0.893 | 0.581 | 1.7E-04 | * | 0.920 | 0.581 | 2.8E-05 | * | 0.920 | 0.574 | 1.6E-05 | * |
|  | MV | Kurtosis | 0.893 | 0.535 | 6.6E-04 | * | 0.920 | 0.535 | 1.1E-04 | * | 0.920 | 0.505 | 6.2E-05 | * |
| **D_2_'** | MV | Median | 0.816 | 0.812 | 7.2E-01 |  | 0.852 | 0.812 | 1.5E-02 | * | 0.852 | 0.843 | 3.1E-01 |  |
|  | MV | Std | 0.816 | 0.658 | 1.2E-01 |  | 0.852 | 0.658 | 3.5E-02 | * | 0.852 | 0.614 | 1.0E-02 | * |
|  | MV | P5 | 0.816 | **0.856** | 2.4E-01 |  | 0.852 | **0.856** | 9.0E-01 |  | 0.852 | **0.865** | 6.5E-01 |  |
|  | MV | P10 | 0.816 | **0.850** | 1.7E-01 |  | 0.852 | 0.850 | 9.3E-01 |  | 0.852 | **0.860** | 7.3E-01 |  |
|  | MV | P25 | 0.816 | **0.849** | 5.4E-02 |  | 0.852 | 0.849 | 8.2E-01 |  | 0.852 | **0.858** | 6.2E-01 |  |
|  | MV | P75 | 0.816 | 0.762 | 1.3E-02 | * | 0.852 | 0.762 | 3.6E-03 | * | 0.852 | 0.817 | 3.0E-02 | * |
|  | MV | P90 | 0.816 | 0.731 | 6.1E-03 | * | 0.852 | 0.731 | 2.0E-03 | * | 0.852 | 0.789 | 2.7E-02 | * |
|  | MV | P95 | 0.816 | 0.710 | 1.5E-03 | * | 0.852 | 0.710 | 5.5E-04 | * | 0.852 | 0.766 | 1.2E-02 | * |
|  | MV | Skewness | 0.816 | 0.563 | 2.8E-03 | * | 0.852 | 0.563 | 6.4E-04 | * | 0.852 | 0.535 | 1.4E-04 | * |
|  | MV | Kurtosis | 0.816 | 0.506 | 2.5E-03 | * | 0.852 | 0.506 | 5.9E-04 | * | 0.852 | 0.541 | 2.9E-03 | * |
| **f_1_'** | MV | Median | 0.662 | **0.663** | 9.8E-01 |  | 0.661 | **0.663** | 9.5E-01 |  | 0.661 | **0.665** | 9.1E-01 |  |
|  | MV | Std | 0.662 | 0.510 | 2.2E-02 | * | 0.661 | 0.510 | 2.2E-02 | * | 0.661 | 0.500 | 2.9E-01 |  |
|  | MV | P5 | 0.662 | **0.681** | 7.5E-01 |  | 0.661 | **0.681** | 7.4E-01 |  | 0.661 | **0.681** | 7.4E-01 |  |
|  | MV | P10 | 0.662 | **0.734** | 1.9E-01 |  | 0.661 | **0.734** | 1.8E-01 |  | 0.661 | **0.734** | 1.8E-01 |  |
|  | MV | P25 | 0.662 | **0.684** | 6.8E-01 |  | 0.661 | **0.684** | 6.6E-01 |  | 0.661 | **0.685** | 6.5E-01 |  |
|  | MV | P75 | 0.662 | 0.645 | 2.8E-01 |  | 0.661 | 0.645 | 2.9E-01 |  | 0.661 | 0.644 | 2.8E-01 |  |
|  | MV | P90 | 0.662 | 0.624 | 2.4E-01 |  | 0.661 | 0.624 | 2.5E-01 |  | 0.661 | 0.626 | 2.8E-01 |  |
|  | MV | P95 | 0.662 | 0.610 | 2.1E-01 |  | 0.661 | 0.610 | 2.2E-01 |  | 0.661 | 0.605 | 1.9E-01 |  |
|  | MV | Skewness | 0.699 | 0.654 | 5.6E-01 |  | 0.698 | 0.654 | 5.5E-01 |  | 0.698 | 0.652 | 5.4E-01 |  |
|  | MV | Kurtosis | 0.699 | 0.569 | 1.2E-01 |  | 0.698 | 0.569 | 1.1E-01 |  | 0.698 | 0.568 | 1.2E-01 |  |
| **f_2_'** | MV | Median | 0.667 | **0.678** | 3.7E-01 |  | 0.666 | **0.678** | 3.7E-01 |  | 0.666 | **0.680** | 3.3E-01 |  |
|  | MV | Std | 0.667 | 0.511 | 2.7E-01 |  | 0.666 | 0.511 | 2.7E-01 |  | 0.666 | 0.520 | 3.2E-01 |  |
|  | MV | P5 | 0.667 | **0.702** | 5.1E-01 |  | 0.666 | **0.702** | 5.1E-01 |  | 0.666 | **0.701** | 5.2E-01 |  |
|  | MV | P10 | 0.667 | **0.699** | 4.3E-01 |  | 0.666 | **0.699** | 4.2E-01 |  | 0.666 | **0.697** | 4.5E-01 |  |
|  | MV | P25 | 0.667 | **0.683** | 5.6E-01 |  | 0.666 | **0.683** | 5.5E-01 |  | 0.666 | **0.681** | 6.0E-01 |  |
|  | MV | P75 | 0.667 | 0.664 | 8.4E-01 |  | 0.666 | 0.664 | 9.0E-01 |  | 0.666 | 0.660 | 6.7E-01 |  |
|  | MV | P90 | 0.667 | 0.646 | 3.1E-01 |  | 0.666 | 0.646 | 3.2E-01 |  | 0.666 | 0.640 | 2.5E-01 |  |
|  | MV | P95 | 0.667 | 0.635 | 3.4E-01 |  | 0.666 | 0.635 | 3.5E-01 |  | 0.666 | 0.629 | 2.8E-01 |  |
|  | MV | Skewness | 0.667 | 0.629 | 5.6E-01 |  | 0.666 | 0.629 | 5.8E-01 |  | 0.666 | 0.633 | 6.1E-01 |  |
|  | MV | Kurtosis | 0.667 | 0.566 | 2.1E-01 |  | 0.666 | 0.566 | 2.3E-01 |  | 0.666 | 0.567 | 2.5E-01 |  |
| **D*'** | MV | Median | 0.563 | **0.605** | 3.6E-02 | * | 0.569 | **0.605** | 8.1E-02 |  | 0.559 | **0.597** | 5.6E-02 |  |
|  | MV | Std | 0.563 | **0.573** | 9.5E-01 |  | 0.569 | **0.573** | 9.8E-01 |  | 0.559 | **0.572** | 9.3E-01 |  |
|  | MV | P5 | 0.563 | **0.727** | 7.2E-03 | * | 0.569 | **0.727** | 9.4E-03 | * | 0.559 | **0.717** | 7.9E-03 | * |
|  | MV | P10 | 0.563 | **0.721** | 4.9E-03 | * | 0.569 | **0.721** | 6.6E-03 | * | 0.559 | **0.710** | 5.4E-03 | * |
|  | MV | P25 | 0.563 | **0.674** | 2.0E-02 | * | 0.569 | **0.674** | 2.8E-02 | * | 0.559 | **0.661** | 3.1E-02 | * |
|  | MV | P75 | 0.563 | 0.541 | 9.6E-02 |  | 0.569 | 0.541 | 3.8E-02 | * | 0.559 | 0.536 | 6.6E-02 |  |
|  | MV | P90 | 0.563 | 0.507 | 7.4E-01 |  | 0.569 | 0.507 | 7.1E-01 |  | 0.559 | 0.511 | 7.7E-01 |  |
|  | MV | P95 | 0.563 | 0.560 | 9.9E-01 |  | 0.569 | 0.560 | 9.6E-01 |  | 0.559 | 0.562 | 9.8E-01 |  |
|  | MV | Skewness | 0.563 | 0.536 | 8.2E-01 |  | 0.569 | 0.536 | 7.8E-01 |  | 0.559 | 0.501 | 6.3E-01 |  |
|  | MV | Kurtosis | 0.563 | 0.529 | 7.8E-01 |  | 0.569 | 0.529 | 7.4E-01 |  | 0.559 | 0.509 | 6.8E-01 |  |

AUC – area under the curve, P – p-value, Incl/Excl – ROIs including/excluding necrosis and scars

**Supplementary table 3.** Comparison of AUC values of the ROC curves obtained from 2D and 3D ROIs (see Table 2) at 1.5 T (a) for n=54 (n=33 malignant, n=21 benign) small lesions with volume size <22.0 cm^3^ (0.9-21.9 cm^3^) and b) for n=55 (n=41 malignant, n=14 benign) large lesions with volume size >22.0 cm^3^ (22.1-1715.7 cm^3^).

a) Small ROIs:

| **Par** | **AUC** | **AUC** | **P** |  | **AUC** | **AUC** | **P** |  | **AUC** | **AUC** | **P** |  |
| --- | --- | --- | --- | --- | --- | --- | --- | --- | --- | --- | --- | --- |
|  | **2D** | **3DG** |  |  | **2D** | **3DA** |  |  | **3DG** | **3DA** |  |  |
| **ROIs including centrally deviating areas** | | | | | | | | | | | | |
| **ADC** | 0.921 | 0.921 | 1.000 |  | 0.921 | 0.913 | 0.742 |  | 0.921 | 0.913 | 0.672 |  |
| **D_1_'** | 0.861 | 0.863 | 0.916 |  | 0.861 | 0.864 | 0.881 |  | 0.863 | 0.864 | 0.913 |  |
| **D_2_'** | 0.789 | 0.812 | 0.438 |  | 0.789 | 0.802 | 0.737 |  | 0.812 | 0.802 | 0.670 |  |
| **f_1_'** | 0.619 | 0.646 | 0.656 |  | 0.619 | 0.795 | 0.045 | * | 0.646 | 0.795 | 0.040 | * |
| **f_2_'** | 0.688 | 0.696 | 0.891 |  | 0.688 | 0.820 | 0.059 |  | 0.696 | 0.820 | 0.022 | * |
| **D*'** | 0.504 | 0.577 | 0.187 |  | 0.504 | 0.567 | 0.409 |  | 0.577 | 0.567 | 0.865 |  |
| **ROIs excluding centrally deviating areas** | | | | | | | | | | | | |
| **ADC** | 0.941 | 0.939 | 0.916 |  | 0.941 | 0.932 | 0.671 |  | 0.939 | 0.932 | 0.505 |  |
| **D_1_'** | 0.882 | 0.887 | 0.678 |  | 0.882 | 0.890 | 0.611 |  | 0.887 | 0.890 | 0.800 |  |
| **D_2_'** | 0.810 | 0.835 | 0.343 |  | 0.810 | 0.812 | 0.935 |  | 0.835 | 0.812 | 0.314 |  |
| **f_1_'** | 0.626 | 0.644 | 0.779 |  | 0.626 | 0.801 | 0.042 | * | 0.644 | 0.801 | 0.029 | * |
| **f_2_'** | 0.714 | 0.703 | 0.821 |  | 0.714 | 0.840 | 0.065 |  | 0.703 | 0.840 | 0.012 | * |
| **D*'** | 0.527 | 0.561 | 0.827 |  | 0.527 | 0.551 | 0.867 |  | 0.561 | 0.551 | 0.864 |  |

b) Large ROIs:

| **Par** | **AUC** | **AUC** | **P** |  | **AUC** | **AUC** | **P** |  | **AUC** | **AUC** | **P** |  |
| --- | --- | --- | --- | --- | --- | --- | --- | --- | --- | --- | --- | --- |
|  | **2D** | **3DG** |  |  | **2D** | **3DA** |  |  | **3DG** | **3DA** |  |  |
| **ROIs including centrally deviating areas** | | | | | | | | | | | | |
| **ADC** | 0.929 | 0.908 | 0.255 |  | 0.929 | 0.899 | 0.202 |  | 0.908 | 0.899 | 0.506 |  |
| **D_1_'** | 0.875 | 0.845 | 0.295 |  | 0.875 | 0.850 | 0.395 |  | 0.845 | 0.850 | 0.649 |  |
| **D_2_'** | 0.892 | 0.855 | 0.453 |  | 0.892 | 0.859 | 0.504 |  | 0.855 | 0.859 | 0.781 |  |
| **f_1_'** | 0.645 | 0.613 | 0.647 |  | 0.645 | 0.592 | 0.460 |  | 0.613 | 0.592 | 0.122 |  |
| **f_2_'** | 0.618 | 0.662 | 0.627 |  | 0.618 | 0.678 | 0.546 |  | 0.662 | 0.678 | 0.652 |  |
| **D*'** | 0.598 | 0.507 | 0.101 |  | 0.598 | 0.503 | 0.655 |  | 0.507 | 0.503 | 0.987 |  |
| **ROIs excluding centrally deviating areas** | | | | | | | | | | | | |
| **ADC** | 0.962 | 0.948 | 0.260 |  | 0.962 | 0.939 | 0.293 |  | 0.948 | 0.939 | 0.481 |  |
| **D_1_'** | 0.923 | 0.890 | 0.344 |  | 0.923 | 0.894 | 0.423 |  | 0.890 | 0.894 | 0.736 |  |
| **D_2_'** | 0.939 | 0.904 | 0.376 |  | 0.939 | 0.895 | 0.308 |  | 0.904 | 0.895 | 0.398 |  |
| **f_1_'** | 0.643 | 0.615 | 0.684 |  | 0.643 | 0.585 | 0.406 |  | 0.615 | 0.585 | 0.035 | * |
| **f_2_'** | 0.617 | 0.685 | 0.413 |  | 0.617 | 0.688 | 0.443 |  | 0.685 | 0.688 | 0.911 |  |
| **D*'** | 0.592 | 0.509 | 0.100 |  | 0.592 | 0.502 | 0.096 |  | 0.509 | 0.502 | 0.761 |  |

AUC - area under the curve, * - marks significant results, P- p-value
